# Translesion polymerase eta both facilitates DNA replication and promotes increased human genetic variation at common fragile sites

**DOI:** 10.1073/pnas.2106477118

**Published:** 2021-11-23

**Authors:** Shyam Twayana, Albino Bacolla, Angelica Barreto-Galvez, Ruth B. De-Paula, William C. Drosopoulos, Settapong T. Kosiyatrakul, Eric E. Bouhassira, John A. Tainer, Advaitha Madireddy, Carl L. Schildkraut

**Affiliations:** ^a^Department of Cell Biology, Albert Einstein College of Medicine, Bronx, NY 10461;; ^b^Department of Molecular and Cellular Oncology, University of Texas MD Anderson Cancer Center, Houston, TX 77030;; ^c^Department of Pediatrics Hematology/Oncology, Rutgers Cancer Institute of New Jersey, Rutgers University, New Brunswick, NJ 08903;; ^d^Robert Wood Johnson Medical School, Rutgers University, New Brunswick, NJ 08903

**Keywords:** common fragile sites, polymerase eta, replication fork pause, non-B DNA, SNP

## Abstract

Common fragile sites (CFSs) are difficult-to-replicate genomic regions that form gaps and breaks on metaphase chromosomes under replication stress. They are hotspots for chromosomal instability in cancer. Repetitive sequences located at CFS loci are inefficiently copied by replicative DNA polymerase (Pol) delta. However, translesion synthesis Pol eta has been shown to efficiently polymerize CFS-associated repetitive sequences in vitro and facilitate CFS stability by a mechanism that is not fully understood. Here, by locus-specific, single-molecule replication analysis, we identified a crucial role for Pol eta (encoded by the gene *POLH*) in the in vivo replication of CFSs, even without exogenous stress. We find that Pol eta deficiency induces replication pausing, increases initiation events, and alters the direction of replication-fork progression at CFS-FRA16D in both lymphoblasts and fibroblasts. Furthermore, certain replication pause sites at CFS-FRA16D were associated with the presence of non-B DNA-forming motifs, implying that non-B DNA structures could increase replication hindrance in the absence of Pol eta. Further, in Pol eta-deficient fibroblasts, there was an increase in fork pausing at fibroblast-specific CFSs. Importantly, while not all pause sites were associated with non-B DNA structures, they were embedded within regions of increased genetic variation in the healthy human population, with mutational spectra consistent with Pol eta activity. From these findings, we propose that Pol eta replicating through CFSs may result in genetic variations found in the human population at these sites.

## Significance

Common fragile sites (CFSs) are normal loci that are genetically unstable under normal and oncogenic replication stress. Pol eta has been proposed to play a key role in CFS replication. Here, we show that in the absence of Pol eta, replication at five specific CFS loci is perturbed, with fork pausing observed at several sites. Sequence analysis showed that certain pause sites are associated with the presence of non-B DNA motifs, while others are not. Importantly, pause sites are located within regions of increased genetic variation in healthy human populations that could be attributed to Pol eta activity. Our data unveil a role for Pol eta in overcoming replication stress, reducing DNA breakage, and promoting genetic variation at CFSs.

Common fragile sites (CFSs) are chromosomal regions that are prone to breakage under replication stress and thus are hotspots for genomic rearrangements implicated in cancer development and progression (reviewed in refs. 1 and 2). Under replication stress, CFS expression has been attributed to four prominent CFS characteristics: 1) late S-phase replication (3); 2) enrichment in structure-prone repetitive DNA sequences (4, 5); 3) having sparse replication origins (6, 7); and 4) being hotspots for DNA:RNA hybrid formation and transcription–replication collisions (8). Based on these findings, it is currently thought that defects in DNA replication are the primary reason for genomic instability that arises at CFSs.

We and others have previously shown that facilitator proteins acting in the replication stress response, DNA repair, and DNA replication can influence DNA breakage at CFSs (9–11). One such class of proteins, the specialized translesion synthesis (TLS) DNA polymerases (Pols), maintain CFS stability and prevent chromosomal breakages at CFSs (12, 13). A key TLS Pol in humans, Pol eta (encoded by the *POLH* gene), was initially identified as the gene mutated in a variant form of Xeroderma Pigmentosum (XP), a genetic disorder characterized by extensive sensitivity to ultraviolet (UV) rays and predisposition to sunlight-induced skin cancer (14). In addition to its well-characterized role in the replication of UV-damaged DNA (15–17), Pol eta has recently been shown to play an important role in maintaining CFS stability (13, 18).

The replicative DNA Pol delta pauses at CFS-associated repeat sequences in vitro (19, 20), and TLS Pols like Pol eta are postulated to exchange with paused Pol delta to complete DNA synthesis at CFS-associated repetitive sequences (21). Notably, Pol eta is recruited to CFS loci upon replication stress and could replicate CFS-associated non-B DNA sequences in vitro (13). However, it has not been shown directly that replication pausing occurs at CFS in vivo more frequently in the absence of Pol eta. Here, we directly show in vivo that replication pausing occurs more often at CFSs in the absence of Pol eta, even in the absence of exogenous stress. Our results establish Pol eta’s importance in the replication of CFS sequences and its specific role in maintaining CFS stability.

Using our powerful locus-specific single-molecule analysis of replicated DNA (SMARD) approach, we previously identified important factors affecting replication programs at genomic regions containing repetitive sequences within rare fragile sites (22, 23), telomeres (24, 25), CFSs (9), and episomal Kaposi sarcoma-associated herpesvirus (26). Here, we used SMARD to identify a critical role for Pol eta at CFS loci, even in the absence of exogenous replication stress. We found that Pol eta is important for facilitating DNA replication at CFSs, even during an unperturbed S phase. In the absence of Pol eta, replication-fork pause and initiation events increase at CFS-FRA16D, previously characterized by us (9), in both lymphoblasts and fibroblasts. Furthermore, in Pol eta-deficient fibroblasts, replication-fork progression is perturbed, as evidenced by increased pausing at CFSs loci known to be the most highly expressed in fibroblasts. An in-depth analysis of the sequences underlying the replication pause sites revealed a positive correlation between structure-forming repetitive sequences at the three most prominent pause sites. Pausing observed outside of repetitive DNA can likely be attributed to regions of DNA:RNA formation or transcription:replication collisions, as we have previously shown (9). In addition, we observed increased genetic variation at pause sites in the healthy human population with mutation spectra consistent with Pol eta activity.

## Results

### Replication Is Perturbed at CFS-FRA16D in Pol Eta-Deficient Lymphoblasts.

**Fig. 1.**
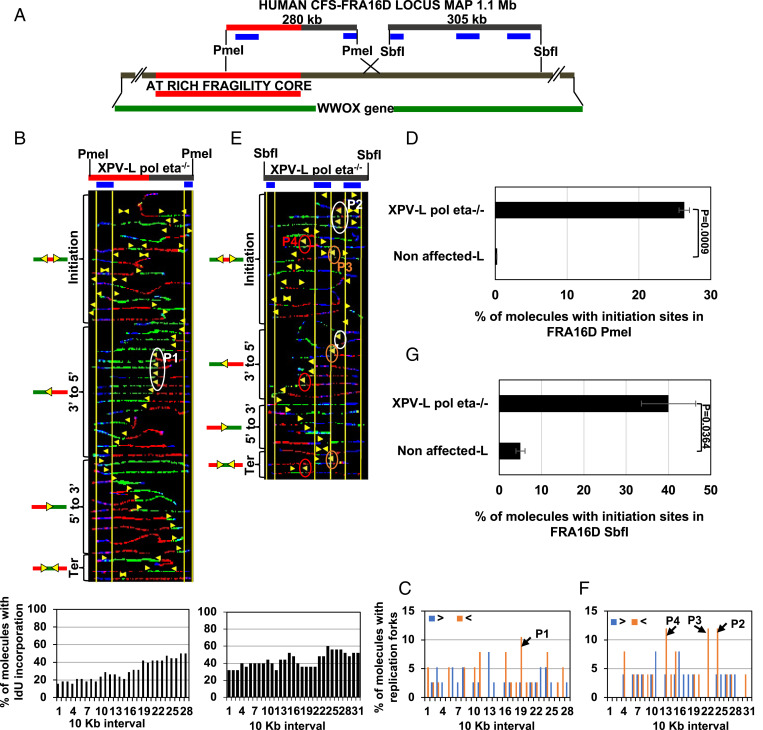
Replication is perturbed at CFS-FRA16D in Pol eta-deficient lymphoblasts. (*A*) A map of the human CFS-FRA16D locus (brown line) overlapping the 1.1-Mb-long *WWOX* gene (green line). The AT-rich fragility core is shown in red. A 280-kb DNA segment and a 305-kb DNA segment generated by cutting with the restriction enzymes PmeI and SbfI, respectively, are shown. The FISH probes used to identify these DNA segments are indicated in blue. (*B*, *Top*) Locus map of the CFS-FRA16D PmeI segment with the location of the FISH probes. (*B*, *Middle*) Photomicrographs of labeled DNA molecules from XPV-L Pol eta^−/−^ lymphoblasts. The yellow arrowheads in each molecule indicate the transition of labeling from 5-iodo-2′-deoxyuridine (IdU) (red) to 5-chloro-2′-deoxyuridine (CldU) (green). Molecules are arranged in the following order: initiation events, forks progressing from 3′ to 5′, forks progressing from 5′ to 3′, and termination events. The white oval (P1) indicates fork pausing. (*B*, *Bottom*) The percentage of molecules with IdU incorporation at each 10-kb interval calculated from molecules above *B*, *Middle* are shown in histograms. (*C*) The percentage of molecules with replication forks at each 10-kb interval in the CFS-FRA16D PmeI, quantified from molecules in *B*. Replication forks progressing from 3′ to 5′ and 5′ to 3′ are denoted by orange < and blue >, respectively. The black arrow indicates the most prominent pause peaks along the CFS-FRA16D PmeI segment and corresponds to the oval in *B*. (*D*) The percentage of molecules with initiation sites in the FRA16D PmeI segment of XPV-L Pol eta^−/−^ lymphoblasts calculated from *B* and nonaffected lymphoblasts calculated from figure 1*C* of ref. 9. Error bars represent mean ± SEM from two independent experiments. (*E*, *Top*) Locus map of the CFS-FRA16D SbfI segment with the location of the FISH probes. (*E*, *Middle*) Photomicrographs of labeled DNA molecules from XPV-L Pol eta^−/−^ lymphoblasts. The yellow arrowheads encircled by white (P2), brown (P3), and red (P4) ovals indicate three different sites where forks paused. (*E*, *Bottom*) The percentage of molecules with IdU incorporation at each 10-kb interval calculated from molecules above *E*, *Middle* are shown in histograms. (*F*) The percentage of molecules with replication forks at each 10-kb interval in the CFS-FRA16D SbfI segment, quantified from molecules in *E*. Replication forks progressing from 3′ to 5′ and 5′ to 3′ are denoted by orange < and blue >, respectively. The black arrows indicate the most prominent pause peaks along the CFS-FRA16D segment and correspond to the ovals in *E*. (*G*) The percentage of molecules with initiation sites in the FRA16D SbfI segment of XPV-L Pol eta^−/−^ lymphoblasts calculated from *E* and nonaffected lymphoblasts calculated from figure 3*B* of ref. 9. Error bars represent mean ± SEM from two independent experiments.

Pol eta is recruited to CFSs in vivo and can efficiently replicate through CFS-associated repetitive sequences in vitro (13, 21). However, whether Pol eta plays a critical role during in vivo DNA replication of CFSs was undetermined. Here, we analyzed the role of Pol eta in the replication of CFSs in vivo by using our unique DNA-replication assay termed SMARD. SMARD reveals replication-fork direction, pausing, initiation, and termination events, as well as the region that is replicated first within a specific genomic locus (27) (*SI Appendix*, Fig. S1 *A* and *B*). The method used for analysis of pausing is depicted in *SI Appendix*, Fig. S1*B*. We analyzed two segments within the CFS-FRA16D locus: a 280-kb PmeI segment containing a portion of the AT-rich fragility core and an adjacent 305-kb SbfI segment (Fig. 1*A*). We previously characterized the replication programs at both of these segments in nonaffected lymphocytes expressing wild-type (WT) Pol eta (9). In nonaffected lymphoblastoid cell lines, replication at the 280-kb PmeI segment was carried out by equal numbers of forks moving in both the 5′ to 3′ and 3′ to 5′ directions, with no prominent replication-initiation events or fork pausing (9).

To test the effect of Pol eta’s absence on the dynamics of DNA replication at CFS-FRA16D, we analyzed a patient-derived Pol eta-deficient XP variant (XPV) lymphoblastoid cell line (XPV-L Pol eta^−/−^). In contrast to nonaffected cells, absence of Pol eta was associated with pausing of replication forks progressing from 3′ to 5′, just before they entered the AT-rich fragility core in the 280-kb PmeI segment (Fig. 1*B*, white oval; and Fig. 1*C*, black arrow, P1). A significant increase in replication-initiation events was also seen in the absence of Pol eta, where ∼26% of molecules showed initiation events at this locus, which does not contain any initiation events in nonaffected cells (Fig. 1 *B* and *D*). The 3′ end of this segment was replicated before the 5′ end in the absence of Pol eta (Fig. 1 *B*, *Bottom*), in contrast to nonaffected cells, where it is not evident that any one end is replicated before the other (9).

In nonaffected lymphoblastoid cells, the 305-kb SbfI segment was replicated predominantly from replication forks progressing from the 3′ to 5′ direction with no prominent pausing or replication-initiation events (9). By contrast, in cells lacking Pol eta, replication at the 305-kb SbfI segment was carried out by an equal number of forks progressing in both the 5′ to 3′ and 3′ to 5′ directions (Fig. 1*E*), with forks pausing in 3′ to 5′ direction (Fig. 1*E*, white, brown, and red ovals; and Fig. 1*F*, black arrows, P2–P4). A substantial increase in replication initiation events was also observed, with ∼40% of molecules showing initiation events, compared to ∼5% in nonaffected cells (Fig. 1 *E* and *G*). It was not evident that any one end of this segment was replicated before the other in the absence of Pol eta (Fig. 1 *E*, *Bottom*), in contrast to nonaffected cells, where the 3′ end was replicated before the 5′ end (9).

Replication-fork stalling can be accompanied by the activation of dormant origins, a cellular response to generate more replication forks to prevent replication slowdown (28, 29). The replication pausing and striking dormant origin response observed at CFS-FRA16D, in the absence of Pol eta, underscores Pol eta’s importance for CFS-FRA16D replication in lymphocytes.

### Complementation with WT Pol Eta Overcomes Replication Perturbation at CFS-FRA16D.

**Fig. 2.**
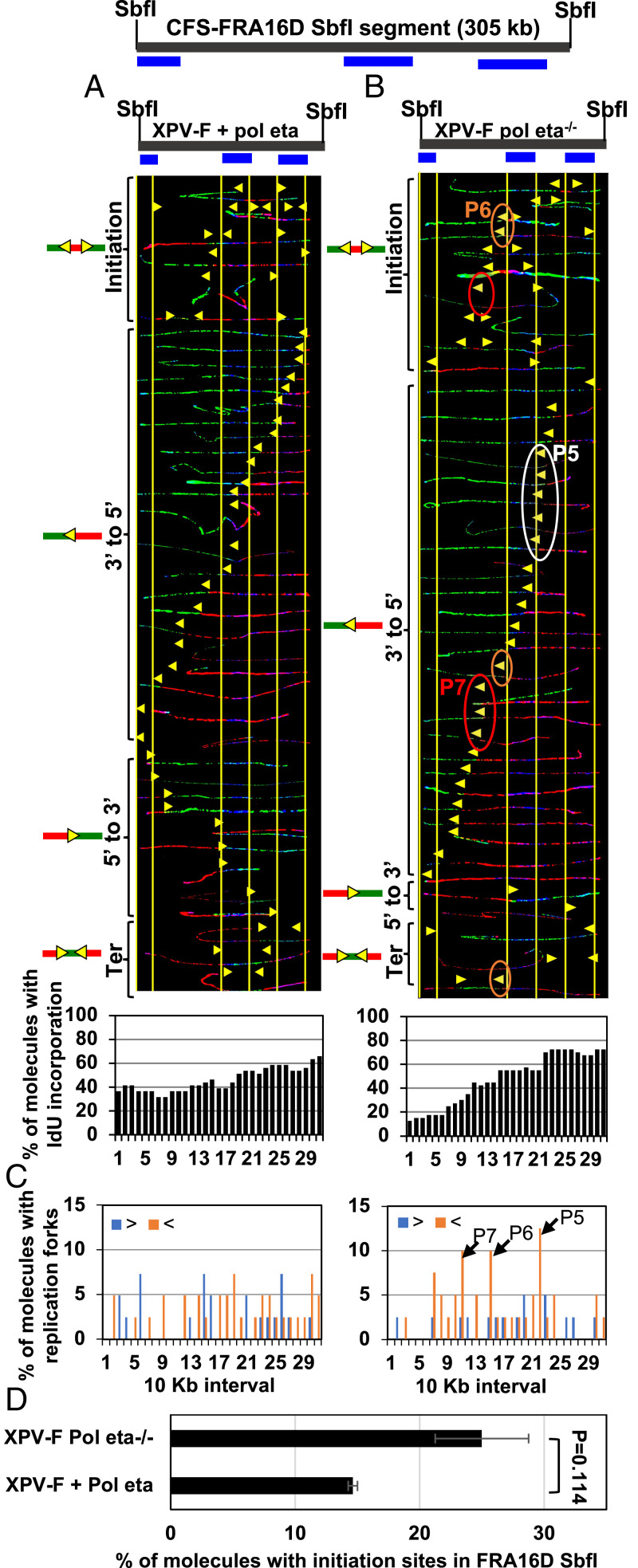
Replication is perturbed at the CFS-FRA16D in Pol eta-deficient fibroblasts. (*A* and *B*, *Top*) Locus map of the CFS-FRA16D SbfI segment with the location of the FISH probes. (*A* and *B*, *Middle*) Photomicrographs of labeled DNA molecules from XPV Pol eta^−/−^ fibroblasts stably complemented with Pol eta (*A*) or not (*B*). Molecules are arranged as in Fig. 1. The yellow arrowheads encircled by white (P5), brown (P6), and red (P7) ovals indicate three different sites where replication forks paused. For each cell line, DNA molecules were photographed and collected from two sets of slides, where the molecules were independently stretched and independently stained with IdU and CldU antibodies (and the probe). Molecules that represented pausing at P5–P7 were independently identified in both sets of slides, showing that pausing molecules do not result from experimental variation due to a stretching or a staining artifact on a particular slide. (*A* and *B*, *Bottom*) The percentage of molecules with IdU incorporation at each 10-kb interval calculated from molecules above (*A* and *B*, *Middle*) are shown in histograms. (*C*) The percentage of molecules with replication forks at each 10-kb interval in the FRA16D SbfI segment, quantified from molecules in *A* and *B*. Replication forks progressing from 3′ to 5′ and 5′ to 3′ are denoted by orange < and blue >, respectively. The black arrows indicate the pause sites (P5–P7) and correspond to the ovals in *B*. (*D*) The percentage of molecules with initiation sites in FRA16D SbfI segments calculated from molecules in *A* and *B*. Error bars represent mean ± SEM from two independent experiments.

To determine whether aberrant replication phenotypes observed in XPV-L Pol eta^−/−^ cells were indeed due to the absence of Pol eta, we next analyzed a pair of isogenic XPV-F Pol eta^−/−^ and WT Pol eta-complemented fibroblasts (XPV-F + Pol eta) (30). We used fibroblasts for our analysis due to the inherent difficulties in complementing lymphocytes, which grow in suspension. We previously found that while the replication program of CFS-FRA16D is different between lymphocytes and fibroblast (9), the locus is fragile in fibroblasts, albeit to a lesser extent than in lymphoblasts (9, 31). Here, we analyzed the replication program of the 305-kb SbfI segment of CFS-FRA16D. Replication forks progressed predominantly in the 3′ to 5′ direction with no prominent pausing in XPV-F + Pol eta cells (Fig. 2*A*). Remarkably, in XPV-F Pol eta^−/−^ fibroblasts, three prominent pause sites were observed (Fig. 2*B*, white, brown, and red ovals; and Fig. 2*C*, black arrows, P5–P7). There was also a decrease in forks progressing in the 5′ to 3′ direction (Fig. 2*B*) in XPV-F Pol eta^−/−^, compared to XPV-F + Pol eta fibroblasts. The 3′ end of the 305-kb SbfI segment of CFS-FRA16D was predominantly replicated before the 5′ end in XPV-F Pol eta^−/−^ fibroblasts, but this was not as evident in XPV-F + Pol eta fibroblasts (Fig. 2 *A* and *B*, *Bottom*). Furthermore, in XPV-F Pol eta^−/−^ fibroblasts, there was an ∼1.7-fold increase in molecules with initiation events at the CFS-FRA16D SbfI segment compared to in XPV-F + Pol eta fibroblasts (Fig. 2*D*). Thus, we detected prominent fork pausing, altered replication-fork direction, and additional activated replication origins at the CFS-FRA16D SbfI segment in the Pol eta-deficient, compared to Pol eta-proficient, fibroblasts. Furthermore, the replication program of the WT Pol eta-complemented fibroblasts at CFS-FRA16D was similar to the program observed in nonaffected fibroblasts (9). These data indicate that complementation with WT Pol eta eliminates replication perturbation at the CFS-FRA16D SbfI segment.

The repetitive nature of the genomic sequence at CFS-FRA16D is a distinctive feature common to both lymphoblasts and fibroblasts. Notably, Pol eta can replicate through repetitive sequences associated with CFSs in vitro (13, 21). Besides reaffirming the importance of Pol eta in facilitating CFS replication, these data support the view that Pol eta contributes to the replication of repetitive genomic sequences of CFS-FRA16D.

### Replication Programs at Fibroblast-Specific Fragile Sites Are Perturbed in Pol Eta-Deficient Fibroblasts.

**Fig. 3.**
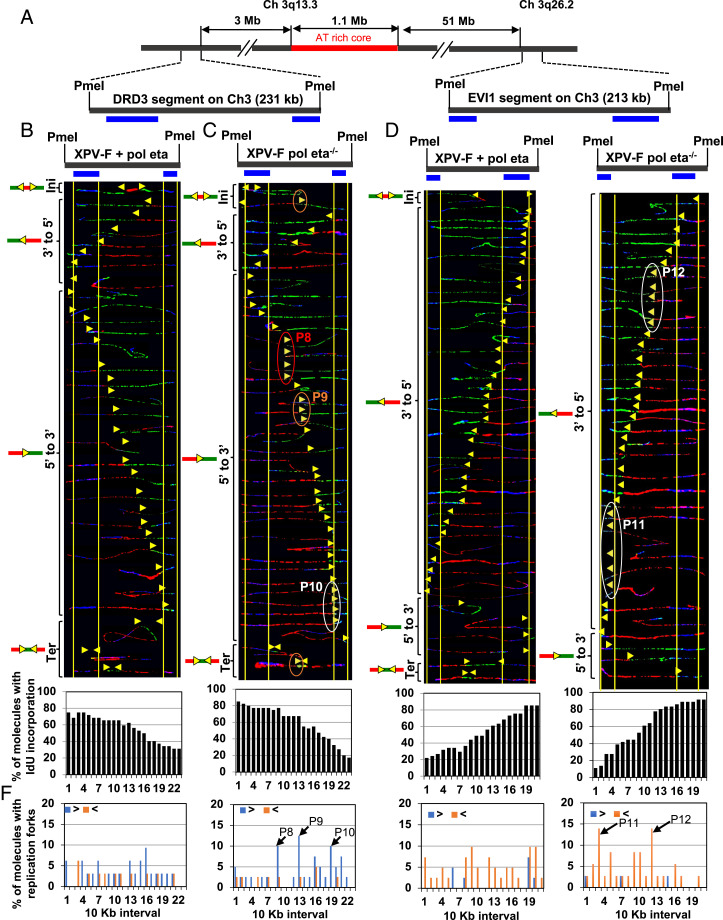
Replication is perturbed at the CFS on chromosome 3q13.3 in Pol eta-deficient fibroblasts. (*A*) Locus map of DRD3 and EVI1 segments with respect to the AT-rich core of the CFS at chromosome 3q13.3. (*B* and *C*, *Top*) Locus map of the DRD3 segment with the location of the FISH probes. (*B* and *C*, *Middle*) Photomicrographs of labeled DNA molecules from XPV Pol eta^−/−^ fibroblasts stably complemented with Pol eta (*B*) or not (*C*). Molecules are arranged as in Fig. 1. The yellow arrowheads encircled by red (P8), brown (P9), and white (P10) ovals indicate three different sites where replication forks paused. As in Fig. 2, molecules that represented pausing at P9–P10 were independently identified in two sets of slides. (*B* and *C*, *Bottom*) The percentage of molecules with IdU incorporation at each 10-kb interval quantified from molecules above (*B* and *C*, *Middle*). (*D* and *E*, *Top*) Locus map of the EVI1 segment with the location of the FISH probes. (*D* and *E*, *Middle*) Photomicrographs of labeled DNA molecules from XPV Pol eta^−/−^ fibroblasts stably complemented with Pol eta (*D*) or not (*E*). Molecules are arranged as in Fig. 1. The white ovals (P11 and P12) indicate replication-fork pausing. As in Fig. 2, molecules that represented pausing at P11–P12 were independently identified in two sets of slides, showing that the detection of pausing does not result from experimental variation. (*D* and *E*, *Bottom*) The percentage of molecules with IdU incorporation at each 10-kb interval quantified from molecules above (*D* and *E*, *Middle*). (*F*) The percentage of molecules with replication forks at each 10-kb interval in the DRD3 and EVI1 segments, quantified from molecules in *B*–*E*. Replication forks progressing from 3′ to 5′ and 5′ to 3′ are denoted by orange < and blue >, respectively. The black arrows indicate the most prominent pause peaks along these segments and correspond to the ovals in *C* and *E*.

**Fig. 4.**
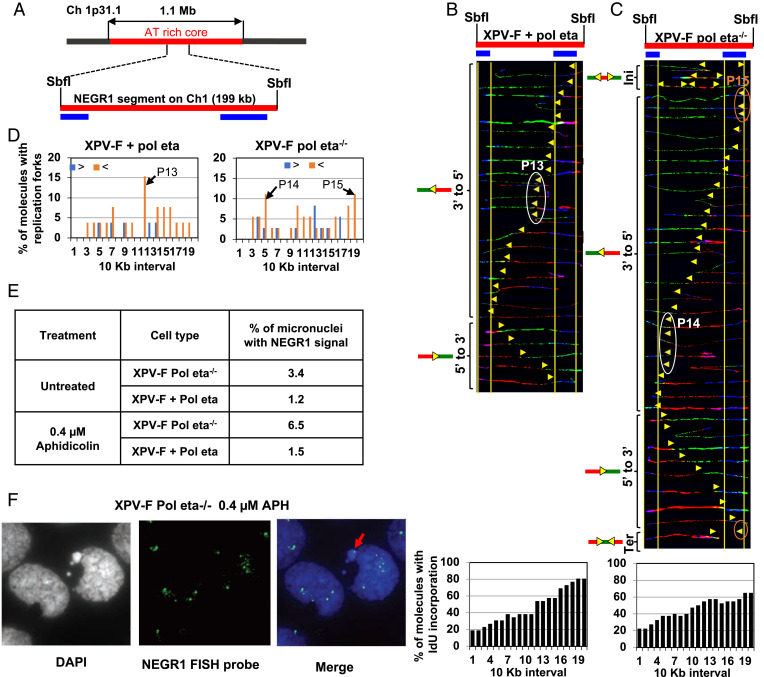
Pol eta deficiency is associated with replication pausing and instability at CFS on chromosome 1p31.1. (*A*) Locus map of NEGR1 with respect to the AT-rich core of the CFS at chromosome 1p31.3. (*B* and *C*, *Top*) Locus map of NEGR1 segment with the location of the FISH probes. (*B* and *C*, *Middle*) Photomicrographs of labeled DNA molecules from XPV Pol eta^−/−^ fibroblasts stably complemented with Pol eta (*B*) or not (*C*). Molecules are arranged as in Fig. 1. The white ovals (P13 and P14) and the brown ovals (P15) indicate replication-fork pausing. As in Fig. 2, molecules that represented pausing at P13–P15 were independently identified in two sets of slides, showing that the detection of pausing does not result from experimental variation. (*B* and *C*, *Bottom*) The percentage of molecules with IdU incorporation at each 10-kb interval quantified from molecules above (*B* and *C*, *Middle*). (*D*) The percentage of molecules with replication forks at each 10-kb interval in the NEGR1 segment, quantified from molecules in *B* and *C*. Replication forks progressing from 3′ to 5′ and 5′ to 3′ are denoted by orange < and blue >, respectively. The black arrows (P13–P15) indicate the most prominent pause peaks along the NEGR1 segment and correspond to the white and brown ovals in *B* and *C*. (*E*) Table representing the percentage of micronuclei with the NEGR1 FISH signal in XPV-F Pol eta^−/−^ or XPV-F + Pol eta cells in the presence or absence of 0.4 µM aphidicolin. At least 2,000 cells were counted for each condition. (*F*) Representative image of micronuclei with NEGR1 FISH signal. The red arrow indicates a micronucleus containing the NEGR1 segment.

A CFS is said to be expressed in a particular cell type if it shows gaps and breaks in metaphase chromosome. CFS expression and instability are cell-type-specific phenomena (32). While CFS-FRA16D is an exception to the rule, it is only moderately fragile in fibroblasts (31). Analysis of FRA16D in fibroblasts, while informative, might not accurately represent the importance of Pol eta to fragile-site stability in fibroblasts. Therefore, we analyzed the replication programs at four gene-containing loci within fibroblast-specific fragile sites located on chromosomes 1 and 3 (Figs. 3*A* and 4*A* and *SI Appendix*, Fig. S2*A*). The regions analyzed included a 213-kb PmeI segment, which contains the EVI1 oncogene (3q26.2); a 231-kb PmeI segment that overlaps with the dopamine receptor D3 (DRD3) gene; a 199-kb SbfI segment that includes the NEGR1 gene, which is located within the fragility core at 1p31.1; and a 296-kb SbfI DNA segment that overlaps part of the NFIA gene (33, 34). While the EVI1 and NEGR1 loci were chosen because they are classic fibroblast-specific fragile sites, the NFIA and DRD3 loci were included due to the high frequencies of chromosomal aberrations observed at these sites and their close proximity to the classic fragile sites located in these chromosomes (35, 36).

Analysis of replication dynamics at these four regions revealed prominent replication-fork pausing at the DRD3 (Fig. 3*C*, red, brown, and white ovals; and Fig. 3*F*, black arrows, P8–P10), EVI1 (Fig. 3*E*, white ovals; and Fig. 3*F*, black arrows, P11–P12), and NFIA (*SI Appendix*, Fig. S2*C*, white oval; and *SI Appendix*, Fig. S2*D*, black arrow P16) segments in XPV Pol eta^−/−^ fibroblasts, but not in the isogenic cells complemented with WT Pol eta. Within the NEGR1 segment, while we observed two pause sites in XPV Pol eta^−/−^ fibroblasts (Fig. 4*C*, white and brown ovals; and Fig. 4*D*, black arrows, P14–P15), we only detected one in the isogenic cells complemented with WT Pol eta (Fig. 4*B*, white oval; and Fig. 4*D*, black arrow, P13). The 5′ end of the DRD3 segment was replicated before the 3′ end in both the XPV-F Pol eta^−/−^ and XPV-F + Pol eta fibroblasts (Fig. 3 *B* and *C*, *Bottom*). The 3′ end of the EVI1 and NEGR1 segments were replicated before the 5′ end in both the XPV-F Pol eta^−/−^ and XPV-F + Pol eta fibroblasts (Fig. 3 *D* and *E*, *Bottom*, and Fig. 4 *B* and *C*, *Bottom*).

Altogether, these results show that Pol eta is required for unperturbed replication at the CFSs expressed in fibroblasts and that its absence causes fork pausing, strengthening the concept that Pol eta facilitates DNA replication at CFSs.

### Absence of Pol Eta Is Associated with Genomic Instability at the NEGR1-CFS Locus on Chromosome 1p31.1.

Upon replication stress, cells can enter mitosis with underreplicated CFSs, despite the activation of dormant origins to rescue replication (reviewed in ref. 37). Therefore, perturbation in DNA replication at CFSs has been associated with incompletely replicated DNA in G2/M, which can be visualized as ultrafine DNA bridges. If left unresolved, these DNA bridges can break and give rise to micronuclei containing fragile-site sequences (38). To understand the consequence of perturbed DNA replication, in the absence of Pol eta, on the stability of the NEGR1 locus, we carried out a fluorescence in situ hybridization (FISH) analysis in micronuclei. We used FISH probes containing the NEGR1 segment sequence, which is located inside the AT-rich core of the CFS at chromosome 1p31.1 (Fig. 4*A*). XPV Pol eta^−/−^ fibroblasts showed an ∼2.8-fold increase in micronuclei with NEGR1 signal compared to isogenic cells complemented with WT Pol eta (Fig. 4 *E* and *F*). When treated with the replicative inhibitor aphidicolin, XPV Pol eta^−/−^ fibroblasts showed an ∼4.3-fold increase in micronuclei with NEGR1 signal, compared to aphidicolin-treated isogenic cells complemented with WT Pol eta. These results suggest that perturbation of replication at the NEGR1 segment, due to the absence of Pol eta, leads to instability, even in the absence of exogeneous stress, and this effect is further exacerbated upon replicative DNA Pol inhibition.

### Non-B DNA Structures Are Associated with Pausing at Certain CFS-FRA16D Regions in the Absence of Pol Eta.

Replicative Pols such as Pol delta pause at repetitive DNA sequences that can form non-B DNA structures (19, 20). In contrast, TLS Pol eta efficiently replicates through these secondary DNA structure-forming sequences (21, 39, 40). Absence of Pol eta led to replication pausing at distinct sites along the CFS regions that we analyzed (Figs. 1 *C* and *F*, 2*C*, 3*F*, and 4*D* and *SI Appendix*, Fig. S2*D*). While pausing at fragile-site loci can be attributed to repetitive DNA-associated secondary structure formation, we have shown that it can also be attributed to the formation of transcription-associated secondary structures called R loops (9). To differentiate between these possibilities, we assessed whether the pause sites we observed in the absence of Pol eta were enriched in non-B DNA-forming motifs.

We analyzed five different types of non-B DNA-forming motifs at each 10-kb interval along the CFS-FRA16D PmeI/SbfI, DRD3, EVI1, NEGR1, and NFIA segments using two methods: a set of custom scripts (41) and the web-based search tool non-B DB version v2.0 (42), thereby employing slightly different criteria for motif identification. The types of non-B DNA-forming motifs analyzed were inverted repeats capable of forming cruciform structures; direct (tandem) repeats, which can give rise to slipped—out-of-register—DNA helices with single-stranded loops; homo(purine⋅pyrimidine) motifs with mirror repeat symmetry (e.g., AGGGAGGxxGGAGGGA) able to form triplex DNA and triplex DNA:RNA hybrids; alternating purine–pyrimidine tracts (e.g., TGTGTGCGCG) that may flip from the right-handed B form to a left-handed Z helix (Z-DNA); and four or more runs of GGG separated by short (1 to 7 nt) spacers, which may form quadruplex (G4) DNA (41). We counted the total number of motifs for each of the five non-B DNA-forming motifs in 10-kb intervals, which is the same resolution as that of replication pause sites determined by SMARD, and compared the numbers of non-B DNA-forming motifs between intervals that contained a pause site and flanking intervals that were devoid of pause sites. We also counted the total number of bases involved in each type of predicted non-B DNA structure, as well as the total number of bases involved in the composite non-B DNA-forming motifs, to assess the relative density between pause-containing and pause-free intervals. Finally, we recorded the longest motifs in both pause-containing and pause-free intervals (*SI Appendix*, Table S1).

**Fig. 5.**
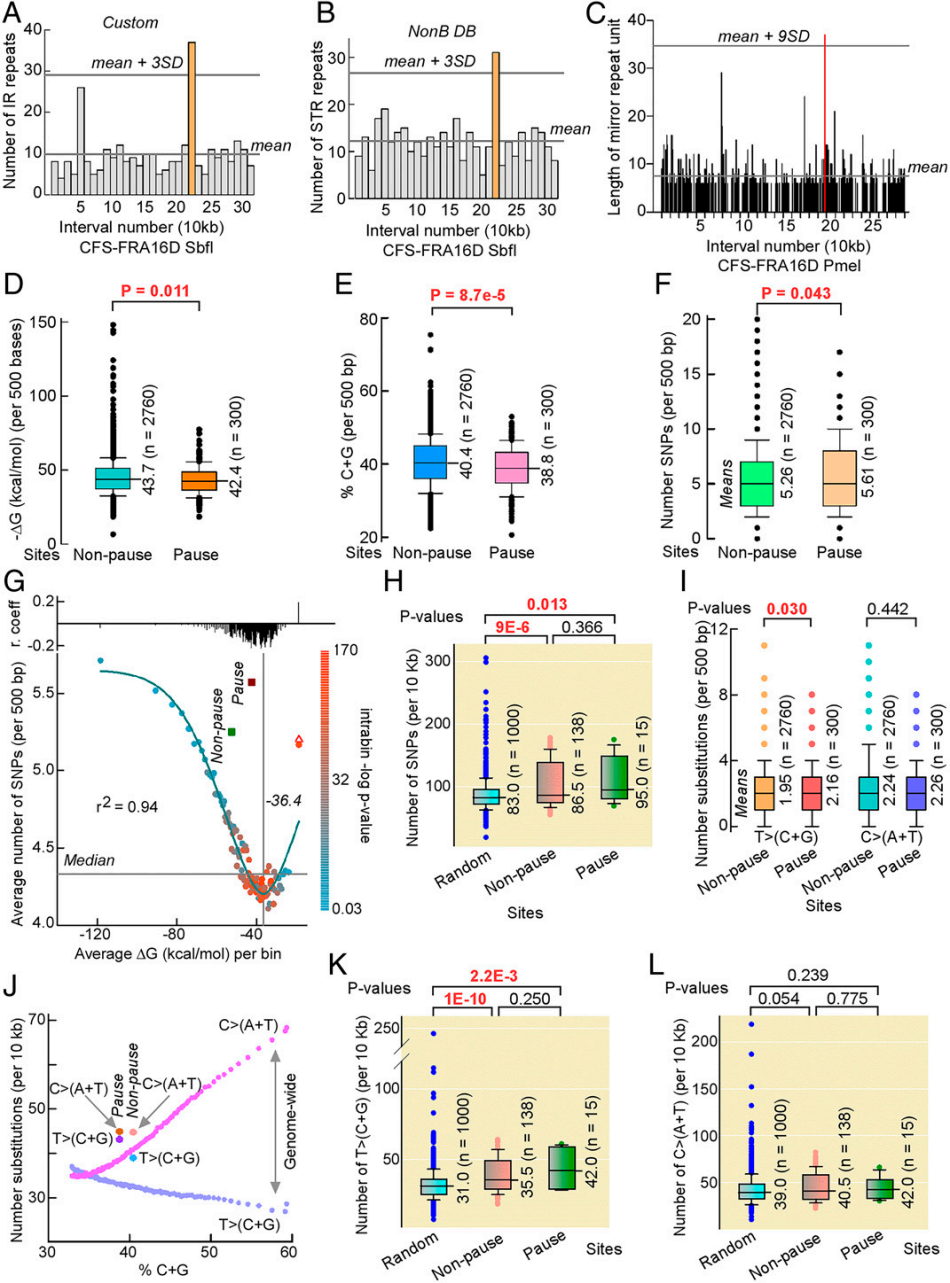
Pausing is linked to non-B DNA structures, low C+G-content, and increased genetic variation in healthy human populations. (*A* and *B*) Bar plot of inverted repeats found by custom scripts (*A*) and simple tandem repeats found by non-B DB (*B*) in each 10-kb interval of the CFS-FRA16D SbfI segment. Orange, interval 22, which contains pause site P3 in Pol eta-deficient lymphoblasts and P5 in Pol eta-deficient fibroblasts; reference lines, mean and mean + 3 SDs for non-B DNA-forming repeats in the total 31 intervals. (*C*) Bar plot of homo(purine⋅pyrimidine) tract lengths with mirror repeat symmetry in each 10-kb interval of the CFS-FRA16D PmeI segment. Lengths are for a single one of each mirror repeat to avoid spacer contributions. The longest homo(purine⋅pyrimidine) tract along CFS-FRA16D PmeI segment is within pause site P1 (red). (*D–F*) Box plots of −Δ*G* (kcal/mol) values of the most stable mfold-predicted structure for each 500-base interval of the combined six CFS segments (*D*), of %C+G for the 500-base intervals comprising the combined six CFS fragments (*E*), and of SNPs per 500 bp in the combined six CFS fragments (*F*). Mean and total intervals are shown. (*G*) Number of SNPs versus Δ*G* genome-wide. Color code shows the −log10 *P* value for the correlation between SNPs and Δ*G* within each data point with regression coefficients (*r*) shown on top. Reference lines, median and curve minimum; red triangle, Δ*G* for the 53,972 reverse sequences of the last data point; green and maroon squares, average SNPs and Δ*G* values for the combined 2,760 nonpause and 300 pause site sequences. (*H*) Box plot of SNP numbers in 1,000 10-kb random sequences from hg38 compared with those from nonpause and pause sites normalized to 10 kb. (*I*) Box plot of SNPs substitutions from TA to CG bps, i.e., T>(C+G), and from CG to TA bps, i.e., C>(A+T), in hg38 at nonpause and pause sites for 500-bp intervals. (*J*) Distribution of T>(C+G) (cyan) and C>(A+T) (fuchsia) SNPs as a function of %C+G genome-wide. Each point contains the same sequences as in *G* (last point was excluded) normalized to 10 kb. Data for nonpause and pause sites are shown. (*K* and *L*) Box plot of T>(C+G) (*K*) and C>(A+T) (*L*) SNPs in 1,000 10-kb random sequences from hg38 compared with those from nonpause and pause sites normalized to 10 kb. Median values do not match mean values in *I* for the same data. *P* values are from Mann–Whitney rank-sum tests.

Both the custom scripts and non-B DB v2.0 showed a significantly higher number of inverted repeats (37 and 46, respectively; Fig. 5*A* and *SI Appendix*, Fig. S3*A*, orange bars), a higher number of bases comprising inverted repeats (513 and 1,413, respectively), and a higher number of bases for all non-B DNA-forming motifs (757 and 1,730, respectively) in interval number 22 (*SI Appendix*, Table S1, red) of the CFS-FRA16D SbfI segment, compared to the flanking 30 intervals. Interval 22 corresponds to pause site P3 in Pol eta-deficient lymphoblasts (Fig. 1*E*) and pause site P5 in Pol eta-deficient fibroblasts (Fig. 2*B*). Similarly, non-B DB v2.0 showed a significantly higher number of simple tandem repeats in interval number 22 (Fig. 5*B*, orange bar) compared to the flanking intervals (*SI Appendix*, Table S1, red).

In the CFS-FRA16D PmeI segment, the longest triplex-forming repeat was located within interval number 19 (Fig. 5*C*, red; 37-bp mirror repeat unit), which corresponded to pause site P1 in Pol eta-deficient lymphoblasts (Fig. 1*B*). The sequence was 75 bp long and contained 15 perfect TTCTT repeats (*SI Appendix*, Table S1), which confer mirror symmetry (palindrome) to the entire 75-bp-long sequence. Overall, our analysis of non-B DNA-forming motifs at pause sites P1–P16 support a potential role for cruciform and triplex structures at pause sites P3/5 and P1, respectively, in the absence of Pol eta. Interestingly, analysis of chromosomal rearrangements in cancer recorded in the Catalogue of Somatic Mutations in Cancer (COSMIC) showed that the junction of an intrachromosomal deletion occurred within P3/P5 in one patient and that other junctions were located within P1 in five patients, one of which (WG04M) mapped within the 75-bp homo(purine⋅pyrimidine) triplex-forming motif (*SI Appendix*, Table S2). In summary, our analysis supports the involvement of non-B DNA structures at P3/P5 and P1 stalled forks, in the absence of Pol eta, as a potential source of chromosomal rearrangements in cancer patients (41). The lack of non-B DNA-forming motifs at the remaining pause sites could be indicative of other factors, such as palindromic [AT]n repeats, thermodynamic stability, and transcription-associated conflicts driving replication pausing at these sites.

### Pause Sites Coincide with Regions of Increased Genetic Variation in Healthy Human Populations.

Given the selected numbers of pause sites that were associated with non-B DNA motifs, we first investigated whether palindromic [AT]n repeats would be enriched at P1–P16 since these motifs were reported to contribute to chromosome fragility (2, 5, 20, 43, 44). There were 48 cruciform-forming repeats with short (0 to 7 bases) loops and stem lengths of at least 12 bp in the combined six restriction segments (CFS-FRA16D PmeI and SbfI, DRD3, EVI1, NEGR1, and NFIA), 20 of which were pure [AT]n repeats (*SI Appendix*, Table S3). However, none of these were found at pause sites P1–P16, in agreement with the non-B search analysis.

We therefore reasoned that other features, such as the thermodynamic stability of imperfect and complex stem–loop structures, would distinguish pause sites from nonpause sites. To this end, we divided the DNA sequences of the six restriction segments into a total of 3,060 500-base intervals and determined the −Δ*G* (kcal/mol) of the thermodynamically most stable global folding using mfold. We found that −Δ*G*s were significantly lower at pause sites than at nonpause sites (Fig. 5*D* and *SI Appendix*, Fig. S3*B*), suggesting that imperfect hairpin-loop structures at pause sites were less stable than those at nonpause sites. Consistent with the postulate that hairpin-loop structures with decreased −Δ*G* values would have lower C+G content, a plot of %C+G content for the same 3,060 intervals confirmed that P1–P16 contained a lower C+G content than nonpause sites (Fig. 5*E* and *SI Appendix*, Fig. S3*C*). Interestingly, we also observed a significantly higher number of TT dinucleotides at pause sites compared to nonpause sites (*SI Appendix*, Fig. S3 *D* and *E*). Notably, TT dinucleotides are the main substrate for the generation of UV-induced cyclobutene pyrimidine dimers and a source of increased mutations in Pol eta-defective cells (45).

Next, we posited that the difference in thermodynamic stability of hairpin-loop structures at pause versus nonpause sites would translate into different mutational rates in the human population. Therefore, we compared the number of single nucleotide polymorphisms (SNPs) recorded in the healthy human population between pause sites and nonpause sites. In both cases, the number of SNPs increased as a function of thermodynamic stability of hairpin-loop structures (*SI Appendix*, Fig. S4*A*), such that the aggregate number of SNPs was higher at pause sites than at nonpause sites (Fig. 5*F*). As expected, the number of SNPs also increased with increasing C+G content at both pause and nonpause sites, although the limited number of observations revealed a statistical difference only at 40 to 45% C+G (*SI Appendix*, Fig. S4*B*). As the small differences in SNPs between pause and nonpause sites could arise from random sampling, we conducted a genome-wide analysis on the relationship between mfold Δ*G* values and SNPs.

Upon splitting the reference human genome into ∼6 million 500-base sequences, computing the lowest Δ*G* for each sequence, and grouping results into 100 bins of increasing Δ*G*, a surprising pattern emerged, whereby the number of SNPs first decreased to a minimum as thermodynamic stability of hairpin-loop structures weakened and then increased as Δ*G* values weakened even further near zero (Fig. 5*G*). Within each bin, SNPs correlated negatively with Δ*G*, except for the last bin, where correlation was positive. For this last bin, the average Δ*G* remained low for the complementary DNA (cDNA) strands, suggesting that these 53,972 genomic sites contain characteristic DNA-sequence composition with poor fold-back, but high mutagenic, potential. Overall, the range in SNP frequencies did not exceed ∼35% between the global minimum and the first maximum; nevertheless, the average number of SNPs at both pause and nonpause sites was 30% and 17% higher than the genome-wide average, respectively. Importantly, the average number of SNPs at pause sites was expected to be lower than at nonpause sites, not higher. This supports the conclusion that, on average, SNPs at pause sites occurred more frequently than expected based on their sequence composition, at rates only seen at the highest end of the spectrum genome-wide.

Yet, if local SNP density is highly variable along the genome, this variability could potentially mask differences with both pause and nonpause sites. Therefore, we selected 1,000 random 10-kb sequences from hg38 and compared the SNP distribution with that from pause and nonpause sites, also normalized to 10 kb. Although in the random set, the range of SNPs exceeded that at fragile sites, the distribution was significantly lower than at nonpause and pause sites, despite the fact that the low number of pause sites somewhat weakened the statistical power (Fig. 5*H*), as expected. Therefore, both genome-wide and randomization studies support the conclusion that P1–P16 are embedded within SNP hypervariable regions in the genome and that their susceptibility to pausing further exacerbates such a variability.

We next decomposed the SNPs into their mutational spectra and noted that, with the exception of C>T transitions, substitutions were higher at pause sites than at nonpause sites (*SI Appendix*, Fig. S4*C*), such that substitutions leading to changes from TA to CG pairs, i.e., T>C + T>G or T>(C+G), in short, were selectively higher at pause sites than at nonpause sites (Fig. 5*I*). Genome-wide, the average number of T>(C+G) and C>(A+T) was near-linearly correlated with %C+G (Fig. 5*J*), as expected; the average values at pause and nonpause sites were all higher than their corresponding genome-wide averages, particularly for T>(C+G) at pause sites, which exceeded expectation by ∼30% (10 SNPs per 10 kb). Finally, decomposing the SNPs into their mutational spectra from the randomization analysis conclusively showed that only the rates of T>(C+G) were affected (Fig. 5 *K* and *L*), proving that the increase in SNPs at our fragile sites was caused specifically by increased mutations at TA base pairs (*SI Appendix*, Fig. S4*D*).

High rates of T>(C+G) are part of the complex single-base mutational signature that has been attributed to *POLH* hyperactivation in cancer genomes (https://cancer.sanger.ac.uk/signatures/sbs/sbs9/) (46–48). Therefore, we conducted an analysis of RNA-seq data from The Cancer Genome Atlas and mutational data from COSMIC, which indicated that *POLH* was overexpressed in 8 of 15 tumor types compared to matched normal tissues (*SI Appendix*, Fig. S5*A*) and that the Pol eta mutational signature (signature 9, SBS9) occurred in tumors affecting most tissues, but especially the pancreas, where almost 50% of patients displayed the signature 9-type single-base substitutions (*SI Appendix*, Fig. S5*B*). In these combined cohorts, the aggregate T>(C+G) single-base substitutions increased from 14.2% in signature 9-negative cancer patients to 20.6% in signature 9-positive cancer patients (*SI Appendix*, Fig. S5*C*).

In summary, P1–P16 are distinguished by their reduced tendency to fold into metastable hairpin-loop structures. They coincide with genomic regions characterized by reduced %C+G content relative to their flanking nonpause regions and increased density of TT dinucleotides. Moreover, they are associated with a greater extent of genetic variation in the healthy human populations than their adjacent nonpause sites, displaying a mutational signature consistent with Pol eta’s error-prone translesion activity.

## Discussion

**Fig. 6.**
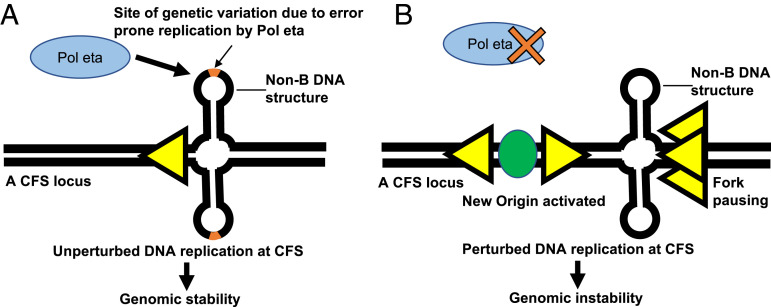
Model depicting the role of Pol eta in the replication of CFS. (*A*) Pol eta facilitates replication of CFS and maintains genomic stability. Pol eta is a translesion Pol that lacks an efficient editing function. Thus, when Pol eta replicates CFS, genetic variation can occur at certain sites, including those containing non-B DNA structures. These sites correspond to the regions of replication-fork pausing in the cells that are deficient for Pol eta (shown in *B*). (*B*) Deficiency of Pol eta leads to perturbed DNA replication and genomic instability at CFS. In the absence of Pol eta, replication forks pause (overlapping yellow arrowheads) at certain sites, including those containing non-B DNA structures. This is accompanied by new origin activation (green oval).

Cells are constantly exposed to both endogenous and exogenous sources of replication stress, which can damage DNA. Single-stranded DNA that is present during replication and transcription is particularly vulnerable to stress and damage. However, cells have inherent safeguards, such as DNA-repair mechanisms, in place to efficiently respond to stress, to prevent genomic instability. How replication stress causes specific types of genetic instability and how local mutation rates vary greatly within individual genomes are key questions for understanding both cell and cancer biology. For example, during transcription, the pretargeting of base excision-repair complexes to open chromatin coincides with local variations in mutation rates across the genome resulting from differential repair of oxidized DNA base damage (49). Likewise, during replication, cells have multiple protective mechanisms in place to safeguard the genome against replicative stress, including ATR-mediated stalled replication-fork restart by the EXO5–BLM complex (50). Notably, the study of CFSs, regions of the genome that are inherently vulnerable to replication stress, has illuminated multiple proteins involved in these mechanisms. Here, we investigated the importance of translesion Pol eta in facilitating replication across CFSs, using the SMARD. Our investigations revealed that Pol eta absence is associated with replicative difficulties at CFSs, characterized by replication-fork pausing and increased initiation events and genomic instability (Fig. 6), likely due to incomplete replication. The increase in initiation events is expected to rescue stalled or collapsed replication forks to ensure replication completion during the S phase, a condition also generated when Pol delta activity is mildly inhibited with low doses of aphidicolin (2, 51).

### Contribution of Structure-Forming Repetitive DNA to Replication Pausing in the Absence of Pol Eta.

Structure-prone sequences are among the important factors that contribute to CFS instability. Replication forks frequently stall at structure-prone A+T-rich sequences (6), which can lead to fork collapse and DNA breakage. TLS Pols can prevent DNA breakage by replicating through such structure-forming sequences (2, 40). Specifically, Pol eta and Pol kappa can exchange with the replicative DNA Pol delta stalled at CFS-associated A+T-rich sequences to complete in vitro DNA replication (21). Here, we found that in the absence of Pol eta, pause sites were observed in both lymphoblasts and fibroblasts at the 305-kb SbfI FRA16D segment, with one pause site (P3/P5) mapping to the same 10-kb interval in both cell types (Figs. 1 and 2). Notably, the genomic sequence of CFS-FRA16D should be common to both lymphoblasts and fibroblasts, although other known factors affecting CFS stability—namely, replication timing, density of origins, and transcription of the locus—might differ. A comprehensive survey of different types of structure-prone sequences known to potentially adopt non-B DNA conformations revealed a clear overrepresentation of perfect inverted repeats and direct repeats in the 10-kb interval comprising P3/P5. The 10-kb interval comprising P3/P5 also harbored more bases potentially involved in non-B DNA structures than all other intervals of the combined six restriction segments analyzed here. In fact, the longest perfect inverted repeat, a 134-base motif, was also located within P3/P5. Interestingly, modeling of a 300-base sequence within P3/P5 containing the 134-bp inverted repeat at the center predicted formation of a large and branched cruciform structure of considerable thermodynamic stability (−Δ*G* value of 59.1 kcal/mol) (*SI Appendix*, Fig. S6*A*) and a perfectly paired 36-bp hairpin with near-zero entropy (*SI Appendix*, Fig. S6*B*). Non-B DB v2.0 also identified other large inverted repeats that were not located within pause sites, the longest of which was a 160-base sequence in interval 14 of the CFS-NEGR1, in which the two arms of the repeats were separated by a large (>20 base) spacer. Modeling of the 300-base sequence containing the 160-base sequence at the center also yielded a highly stable structure (−Δ*G* value of 102.7 kcal/mol); yet, numerous mismatches and large capping loops (*SI Appendix*, Fig. S6*C*) would increase free energy of loop closure and decrease the propensity of hairpin formation (52). Although correlative, these data support the model proposed by Bergoglio et al. (13), who used in vitro primer extension assays to show that Pol eta, but not Pol delta, is able to synthesize through single-stranded templates that fold into duplex hairpins at sites of inverted repeats, as well as templates that contain other sequences that deviate from canonical B-form DNA, such as A-tracts. Therefore, our results support and extend a current hypothesis in the field: A genomic environment with significant secondary structure forming repetitive DNA poses a challenge to processive replicative Pols, and Pol eta has evolved to assist in this challenge.

### Contribution of Transcription-Associated Secondary Structures to Replication Pausing in the Absence of Pol Eta.

The role of DNA secondary structures in pausing is further substantiated by the presence of a long (75 bp), but not unique, homo(purine⋅pyrimidine) track with mirror repeat symmetry in interval 19 of the CFS-FRA16D PmeI segment, which corresponds to pause site P1. Long homo(purine⋅pyrimidine) tracks with mirror repeat symmetry can form stable triplex (H-DNA) structures, which could hinder replication-fork progression (53–57). These sequences occur frequently in introns of large genes, most of which display selective expression in the brain (58). Wang et al. (59) reported an increase in double-strand breaks in such large and transcribed genes in neuronal progenitor cells of macrocephalic autism spectrum disorder toddlers, with proximity ligation assays supporting the notion that these breaks occur at sites of conflicts between converging transcription and replication. Thus, it will be of interest to assess whether DNA:RNA triplexes form in the *WWOX* gene within the 280-kb PmeI segment of CFS-FRA16D and whether Pol eta acts in resolving such conflicts.

At another gene, which encodes the D3 subtype of dopamine receptors (DRD3), two similar-length homo(purine⋅pyrimidine) tracts (83 and 80 bp) found in interval 17 were not associated with pause sites in Pol eta-deficient fibroblasts (*SI Appendix*, Table S1). This can be attributed to the fact that *DRD3*’s expression is restricted to the limbic areas of the brain, in contrast to the 1.1-Mb *WWOX* gene (at CFS-FRA16D) that is transcribed in all tissues (https://gtexportal.org/home/gene/WWOX). Given that gene expression is a critical contributor to CFS instability (8), pause site P1 may be a site of RNA:DNA triplex structures (*SI Appendix*, Fig. S7). RNA:DNA triplex structures, like R loops, may create topological conflicts between converging RNA Pol II and DNA Pol complexes (59–62), which would be absent in the DRD3 segment. Accordingly, transcription of the CFS-FRA16D–associated *WWOX* gene proceeds in the 5′ to 3′ direction (https://www.ncbi.nlm.nih.gov/gene/51741), which is opposite to the direction of fork progression during replication (3′ to 5′) in the P1 pause site. This supports the notion that a collision between converging transcription and replication could occur, leading to replication pausing at P1, which merits consideration for Pol eta involvement in future studies.

### Correlation between Pol Eta-Specific Pause Sites, Repetitive DNA, and POLH Mutational Signature.

SMARD’s ability to detect pause sites within narrow genomic regions was crucial to revealing the increase in genetic variation within the healthy human population at Pol eta-dependent replication pause sites. That this increase in genetic variation at pause sites may be attributed to Pol eta activity is supported by several observations. First, CFSs are relatively A+T-rich, as shown here and in previous studies (reviewed in ref. 2), and Pol eta’s error rate is higher when the base pair preceding misinsertion is T:A or A:T than when it is C:G or G:C (63). Second, an analysis of mutation spectra at somatic hypermutation hotspots within immunoglobulin genes indicated preferred mutation at WA (W is A or T and includes the TT:AA substrate enriched at P1–P16) sequences, displaying strong correlation with Pol eta activity on model substrates (64). Third, in XPV patients, mutations at immunoglobulin variable genes are skewed toward changes at G:C base pairs (65). Fourth, tumor samples overexpressing *POLH* display a characteristic mutational signature known as “signature 9,” characterized by a preponderance of T>C and T>G substitutions (48). Importantly, this mutational signature 9 is also revealed here at Pol eta-dependent pause sites in the context of germline variation, supporting robust Pol eta activity within CFSs.

### Mechanistic Basis for Pol Eta’s Role in CFS Replication.

Whereas replicative DNA Pols replicate DNA with high speed and fidelity, they cannot replicate past DNA lesions and stalled forks. Y-family DNA Pols, such as Pol eta and kappa, have reduced stringency from more open active sites that can accommodate bulky lesions and allow replication through damaged sites (66). In particular, Pol eta is unusual in efficiently extending DNA synthesis from D-loop recombination intermediates, in which an invading strand serves as the primer aided by RAD51 (67). Notably, this preference seems evolutionarily important, as it was codiscovered in the *Escherichia coli* Pol eta homolog Pol V (68). Indeed, the flexible Poly(dA:dT)-rich DNA sequences often found at CFS pause sites have a high capacity for DNA looping (69). Thus, our data on Pol eta-dependent pausing at specific CFSs and the association of pause sites with increased human germline variation, which could be attributed to Pol eta activity, suggest a specific function of Pol eta in the replication of stalled forks at CFSs, possibly by D-loop extension. It will therefore be interesting to see if RAD51 is associated with Pol eta in the DNA replication at CFSs.

In the absence of Pol eta, replication forks progressed beyond the pause site in all DNA segments we analyzed. This observation suggests that pause sites P1–P16 are temporary barriers that may be resolved, perhaps by other TLS Pols, such as Pol kappa. Thus, it would be of interest to analyze functional redundancy between Pol eta and other TLS Pols in the replication of CFSs.

### Implications of Pol Eta Deficiency to CFS Instability and Cancer.

Our comprehensive bioinformatic analysis of the distribution of non-B DNA-forming repeats at the CFSs examined here supports a role for non-B DNA structures in blocking replicative Pols in the absence of Pol eta, reflecting the likely interplay of multiple factors at CFSs. The human genome contains tens of millions of sequences that can form non-B DNA structures (42). These sequences can have a negative role in blocking replication forks, resulting in pausing. Genomic regions with a high density of stable secondary structures, such as P3/5, may constitute mechanical obstacles to Pol progression and are expected to require TLS by Pol eta. Thus, in the absence of Pol eta in XPV cells, there will be pausing that we observe at P3/5. However, most of the pause sites we observed in the absence of Pol eta are not associated with the presence of non-B DNA. Instead, they are characterized by DNA sequences with a reduced tendency to fold into metastable hairpin-loop structures, reduced C+G content, and increased density of TT dinucleotides. Importantly, they furthermore coincide with regions that have increased Pol eta-associated genetic variation among the human population. This suggests that the pause sites that we observed in the absence of Pol eta are more prone to mutation than nonpause sites. Unlike other XP mutants, fibroblasts from XPV patients deficient in the *POLH* gene display normal unscheduled DNA synthesis and recovery of RNA synthesis, but manifest with reduced recovery of DNA synthesis upon exposure to UV radiation (70). Pol eta-null mice display increased DNA damage and an exacerbated DNA-damage response in the adipose tissue, which is partially reversed upon treatment with antioxidants, such as *N*-acetylcysteine (71). These observations suggest that DNA damage arising from endogenous oxidation may require Pol eta for its efficient removal, and it is plausible that pause sites are intrinsically more prone to DNA damage or hinder DNA damage recognition from relevant DNA-repair enzymes such as HMCES, which can shield abasic sites from translesion Pols (72).

Collectively, our data show that Pol eta facilitates replication of CFS loci, thereby revealing a general role for this Pol in response to replication stress at CFSs. We found that some CFS DNA sequences that correspond to certain pause sites in the absence of Pol eta could form non-B DNA structures. Yet, more frequently, these pause sites correspond to DNA sequences that have a reduced tendency to form stable hairpin structures. Moreover, we uncovered an association of these pause sites with regions that have an increased genetic variation in the healthy human populations with mutational spectra linked to Pol eta activity. Going forward, defining the genetic interactions protecting CFSs and other non-Watson–Crick structure-forming DNA sequences will bring new insights into biology and into cancer treatment by informing possible synthetic lethal interactions and vulnerabilities associated with cancers. Currently, our findings decipher the role and mechanism underlying the protection of these unique CFS sequences to enhance and expand our understanding of the role of Pol eta in responding to replication stress and in promoting heritable genetic variation at CFSs.

## Materials and Methods

For additional materials and methods, see also *SI Appendix*.

### Cell Culture.

The XPPHBE (Coriell Cell Repository no. GM02449) Epstein–Barr Virus-transformed XPV lymphoblast cell line (XPV-L Pol eta^−/−^) was grown in Roswell Park Memorial Institute medium supplemented with 15% fetal bovine serum (FBS) and 1% penicillin and streptomycin. The XP30RO SV40-transformed XPV fibroblast cell line (XPV-F Pol eta^−/−^) was grown in Dulbecco’s Modified Eagle Medium/F12 medium (Gibco) supplemented with 15% FBS and 1% penicillin and streptomycin. XPV fibroblasts stably complemented with WT Pol eta (XPV-F + Pol eta) were generated by complementing the XPV-F Pol eta^−/−^ cell line with the Pol eta cDNA [pcDNA 3.1 zeo(−)] and selected in 100 µg/mL Zeocin (30). No detectable Pol eta protein expression was observed in the XPV-F Pol eta^−/−^ cell line, but a significantly high level of Pol eta protein expression was detected in the XPV-F + Pol eta cell line (30). The XPV-F Pol eta^−/−^ and XPV-F + Pol eta cell lines were provided by Kristin A. Eckert, Pennsylvania State University College of Medicine, Hershey, PA.

## Supplementary Material

Supplementary File

## Data Availability

The custom scripts used in this study have been deposited at GitHub (https://github.com/abacolla/nonB-DNA) and will be accessible upon publication (73). All study data are included in the article and/or supporting information.
