# Supplementary material for: Translesion polymerase eta both facilitates DNA replication and promotes increased human genetic variation at common fragile sites
Source: Proc Natl Acad Sci U S A. 2021 Nov 23;118(48):e2106477118. doi: 10.1073/pnas.2106477118 (PMC8640788; doi:10.1073/pnas.2106477118)
Supplement: Supplementary File [file pnas.2106477118.sapp.pdf]

PNAS
www.pnas.org

Supplementary Information for

Translesion polymerase eta both facilitates DNA replication and promotes increased human genetic variation at common fragile sites

Shyam Twayana, Albino Bacolla, Angelica Barreto-Galvez, Ruth B. De-Paula, William C. Drosopoulos, Settapong T. Kosiyatrakul, Eric E. Bouhassira, John A. Tainer, Advaitha Madireddy, and Carl L. Schildkraut

Corresponding authors:

Carl L. Schildkraut
Email: carl.schildkraut@einsteinmed.org

Advaitha Madireddy
Email: advaitha.madireddy@rutgers.edu

This PDF file includes:

SI Materials and Methods
Fig. S1 to S7
Captions for Tables S1 to S3
Table S4

Other supplementary materials for this manuscript include the following:
Tables S1 to S3

SI Materials and Methods

SMARD.

SMARD was carried out as previously described (1-3). Briefly, exponentially growing cells were sequentially pulsed with 30 μM IdU (MP Biomedicals) for 4 hr followed by 30 μM CldU (MP Biomedicals) for 4 hr. Following pulsing, cells were suspended in PBS at a concentration of 3 x 10^7 cells per mL. Equal volume of 1% molten Incert agarose (Lonza) in PBS was added. The resulting cell suspension in 0.5% Incert agarose was poured into wells of a chilled plastic mold to make plugs of size 0.5 cm x 0.2 cm x 0.9 cm, each containing 10^6 cells. Cells in the plug were lysed at 50°C in a buffer containing 1% n-lauroylsarcosine (Sigma-Aldrich), 0.5 M EDTA, and 0.2 mg/mL proteinase K (Invitrogen). Plugs were rinsed in TE and washed with 200 μM phenylmethanesulfonyl fluoride (PMSF) (Sigma-Aldrich). Genomic DNA in the plugs was digested with *PmeI* or *SbfI* (New England Biolabs) overnight at 37°C. Digested genomic DNA was cast into 0.7% SeaPlaque GTG agarose (Lonza) gel and DNA separated by pulsed field gel electrophoresis (PFGE) using a CHEF-DRII system (Bio-Rad). Specific CFS DNA segments in gel slices from pulsed field electrophoresis gel were identified by PCR using specific primers (Table S4). Gel slices were melted and DNA in the gel solution was stretched on a microscopic slide coated with 3-aminopropyltriethoxysilane (Sigma), denatured with sodium hydroxide in ethanol, fixed with glutaraldehyde and hybridized overnight with biotinylated DNA FISH probes at 37°C in a humidified chamber. Biotinylated DNA FISH probes were made from specific fosmids (Table S4). Following hybridization, slides were blocked with 3% BSA for at least 20 min and incubated with the avidin Alexa Fluor 350 (Invitrogen Molecular Probes, Cat # A-11236). This was followed by two rounds of incubation with the biotinylated anti-avidin D (Vector laboratories, Cat # BA-0300) for 20 min followed by the avidin Alexa Fluor 350 for 20 min. Slides were then incubated with an antibody specific for IdU; a mouse anti-BrdU antibody (Becton Dickinson, Cat # 347580), an antibody specific for CldU; a rat monoclonal anti-Brdu antibody (Abcam, Cat # Ab6326), and the biotinylated anti-avidin D for one hr .This was followed by incubation with secondary antibodies: Alexa Fluor 568 goat anti-mouse IgG (H+L) (Invitrogen, Cat # A-11031), Alexa Fluor 488 goat anti-rat IgG (H+L) (Invitrogen, Cat # A-11006), and the avidin Alexa Fluor 350 for one hr. Slides were rinsed in PBS with 0.03% IGEPAL CA-630 after each incubation. After the last rinse in PBS/CA-630, coverslips were mounted on the slides with Prolong gold antifade reagent (Invitrogen). Zeiss fluorescent microscope and IP Lab software (BD) were used to capture images of IdU/CldU incorporated DNA molecules. Images were processed using Adode Photoshop and aligned according to FISH probes pattern using Adobe Illustrator. DNA molecules were obtained from two independently photographed sets of slides where the molecules were independently stretched and independently stained with IdU and CldU antibodies (and the probe). The average number of DNA molecules in each SMARD profile was calculated to be 33.

In SMARD, two or more FISH probes made from fosmids with DNA sequences homologous to specific regions of the DNA segment of interest were used. The probes labeled with biotinylated nucleotides are hybridized and detected using blue fluorescence (Alexa Fluor 350) on the DNA molecule of interest. The probes provide a consistent scale providing a resolution of 5 kb. The asymmetric pattern of the FISH probes is used to determine the orientation of the DNA molecule. FISH probes provide a reference location with a known sequence in each DNA molecule. The molecules of interest are photographed, and the positions of the FISH probes are used to align all the molecules since these probes can be detected in each molecule. To identify pause sites, DNA segments are divided into 10 kb intervals. Pausing is defined to occur when forks were clustered within a 10 kb interval in 10% or more of the molecules. This is shown in the histograms depicting the % of molecules with replication forks at each 10 kb interval (see schematic in Fig.S1B). The 5 kb sequence resolution provided by SMARD as indicated above allows us to confidently locate pause sites within 10 kb intervals.

A profile depicting the region that is replicated first within DNA segment analyzed by SMARD is obtained by plotting the percentage of IdU (first label) incorporation at each 10 kb interval along the DNA segment. The 10 kb intervals that have the highest percentage of molecules with IdU

incorporation are those that on average replicate first within that DNA segment. Replication progressing in only one direction (5´ to 3´ or 3´ to 5´) for most of the DNA segment is represented by a progressive decrease in % IdU incorporation from one end to the other along the segment.

Micronuclei Assay.

Exponentially growing cells were treated with or without 0.4 µM aphidicolin for 16 hrs. Cells were collected by trypsinization and spun on a microscopic slide using Cytocentrifuge (850 rpm, 4 min, StatSpin Cytofuge 2). Slides were treated with methanol acetic acid (3:1) solution at RT for 10 min. Following pretreatment with RNase A, slides were dehydrated by dipping serially in 70%, 95% and 100% ethanol. Samples were denatured in 70% formamide 2x SCC at 75°C for five min. Denaturation of samples was stopped by dipping slides in ice cold 70% ethanol followed by dipping in ice cold 95% and 100% ethanol. Human cot-1 DNA and salmon sperm DNA was added to biotin labeled FISH probe made from BAC clone (RP11-349D17) for NEGR1 segment and denatured at 75 ^{o}C for five min followed by incubation at 37°C for one hr. Slides were then hybridized with denatured FISH probes at 37°C overnight in a humidified chamber. Biotin labeled FISH probe was detected using Alexa Fluor 488-conjugated Avidin (1:10,000, Invitrogen, Cat # A21370). Slides were mounted using Prolong Gold containing DAPI (Invitrogen).

Bioinformatic analyses.

The non-B DNA Motif Search Tool at https://non-B-abcc.ncifcrf.gov/apps/site/default was used to search for direct repeats, simple tandem repeats, inverted repeats, Z-DNA-forming repeats and G4 DNA-forming motifs. Custom scripts (4) were used to retrieve inverted repeats, direct (tandem) repeats, triplex-forming repeats, Z-DNA-forming repeats and G4 DNA-forming sequences. Visualization for the folding of 300-bases into local hairpin-stem loops and entropy values were obtained from RNAfold (http://rna.tbi.univie.ac.at//cgi-bin/RNAWebSuite/RNAfold.cgi). To assess the minimum energy attained by fold-back structures within 300 or 500-base intervals we used mfold/3.6. The mfold program was run on an HPC cluster in a parallel environment using the message passing interface (MPI) and custom C and Bash scripts. To run mfold genome-wide we first divided the hg38 assembly into 6,176,502 500-bases non-overlapping but contiguous intervals and used twoBitToFa (http://hgdownload.soe.ucsc.edu/admin/exe/linux.x86_64/) to extract their genomic sequences. Files containing ~20,000 of these fasta records were then processed by mfold in parallel. After discarding any record with gaps or "Ns", the total number of informative sequences was 5,248,299. A custom Bash script was also used to compute the percent C+G content and non-overlapping TT dinucleotides on either strand on 500-base intervals. The list of SNPs corresponded to dbSNP build 151 and was downloaded from the University of Santa Cruz genome browser at (http://hgdownload.soe.ucsc.edu/goldenPath/hg38/database/). The number of SNPs was computed for the same 500-base intervals used for the mfold and other analyses and averaged over pause sites versus non-pause sites. To retrieve a list of 1000 random 10 kb sequences we used bedtools with the following parameters: bedtools random -l 10000 -n 1500 -seed 17452 -g ../ref/chrSize_hg38 > random_sourceFile to obtain an initial set of 1500 intervals and used twoBitToFa to extract their DNA sequence. We then selected 1000 of these after excluding the Y chromosome and any record with gaps or "Ns". Statistical significance was assessed using non-parametric Mann-Whitney rank sum tests after testing for normality with Shapiro-Wilk tests or Welch's t-tests, as specified. Median values are shown unless indicated otherwise. Signature 9 mutation data in cancer were downloaded from COSMIC at (https://cancer.sanger.ac.uk/cosmic/signatures). Cancer genomic rearrangement junction data were obtained from COSMIC, release 92, file CosmicBreakpointsExport.tsv. *POLH* gene expression data in cancer were from The Cancer Genome Atlas (TCGA) and were obtained through the TCGA Assembler utility (https://github.com/compgenome365/TCGA-Assembler-2) and analyzed using custom scripts. Statistical comparisons between tumor and matched normal controls were preformed using Wilcoxon tests. Only tumor/normal pairs with at least 10 normal samples were included.

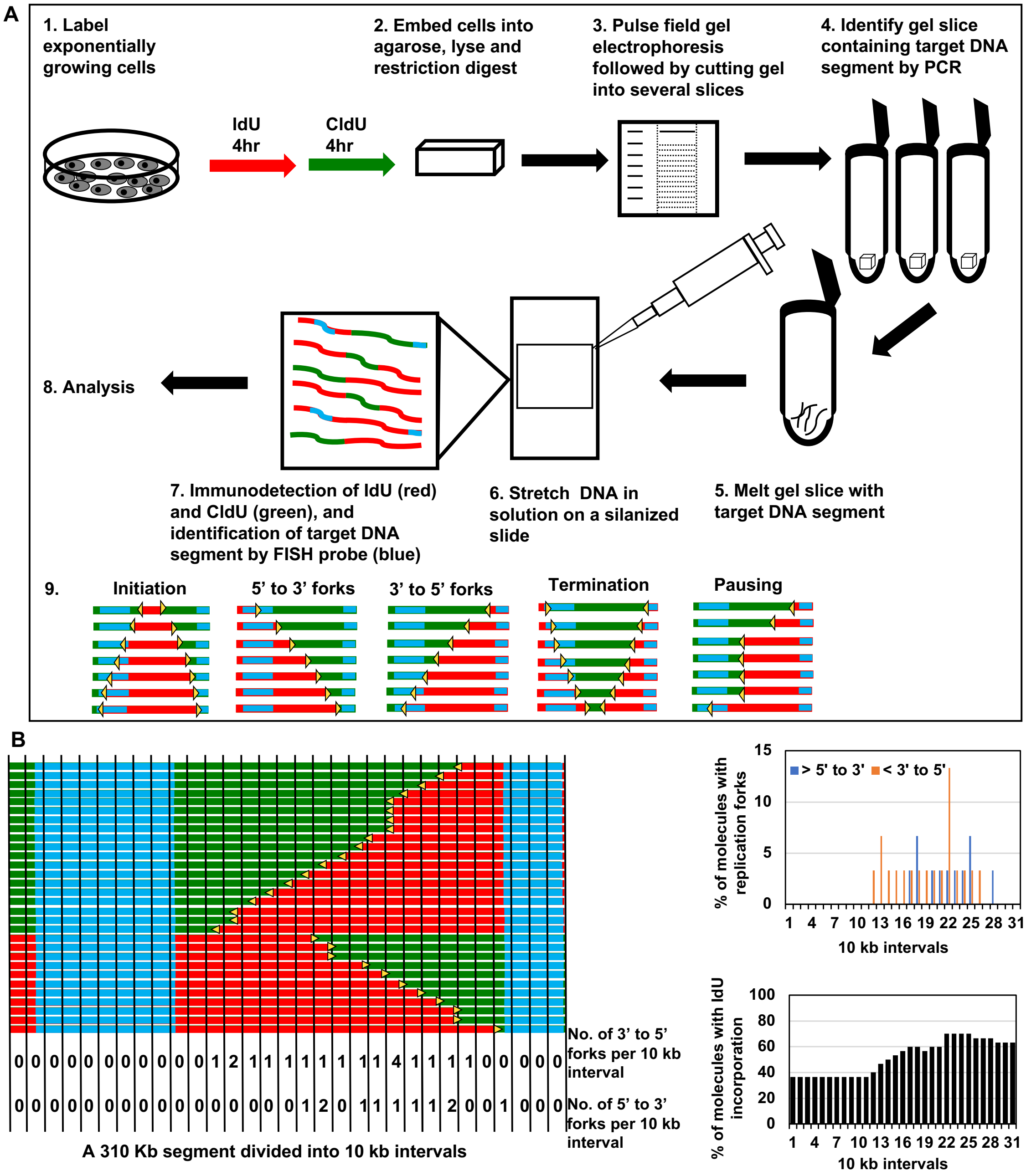

A
1. Label exponentially growing cells
2. Embed cells into agarose, lyse and restriction digest
3. Pulse field gel electrophoresis followed by cutting gel into several slices
4. Identify gel slice containing target DNA segment by PCR
IdU 4hr
CldU 4hr
8. Analysis
7. Immunodetection of IdU (red) and CldU (green), and identification of target DNA segment by FISH probe (blue)
6. Stretch DNA in solution on a silanized slide
5. Melt gel slice with target DNA segment
9.
Initiation
5' to 3' forks
3' to 5' forks
Termination
Pausing
B
0 0 0 0 0 0 0 0 0 0 0 1 2 1 1 1 1 1 1 1 1 4 1 1 1 1 0 0 0 0 0
No. of 3' to 5' forks per 10 kb interval
0 0 0 0 0 0 0 0 0 0 0 0 0 0 0 0 1 2 0 1 1 1 1 1 2 0 0 1 0 0 0
No. of 5' to 3' forks per 10 kb interval
A 310 Kb segment divided into 10 kb intervals
% of molecules with replication forks
> 5' to 3'
< 3' to 5'
10 kb intervals
% of molecules with IdU incorporation
10 kb intervals

Fig. S1. Schematic of single molecule analysis of replicated DNA (SMARD).

A) Schematic representation of different steps of SMARD.1) Exponentially growing cells are sequentially pulsed with two halogenated nucleosides (IdU and CldU) to label replicating DNA. 2) Pulsed cells are lysed in agarose plugs and genomic DNA digested with a restriction endonuclease to produce 100–600 kb segments. 3-4) DNA is separated by pulsed field gel electrophoresis (PFGE) and the target segment identified by PCR. 5-6) A gel slice containing the target segment is melted and the DNA in solution is stretched on silanized slides. 7-8) Halogenated nucleosides are then detected by immunostaining. Biotinylated FISH probes (blue) identify the molecules of interest and are used to align the images of molecules to produce a composite replication profile. 9) Molecules with a red tract flanked by green tracks indicate initiation, molecules with a red track followed by green or vice versa indicate forks progressing in a single direction, molecules with a green tract flanked by red tracks indicate termination, and several molecules with a transition from red to green at the same site indicate pausing. The complete details are in Methods and (1-3).

B) Schematic representation of pausing quantification and replication profile. Left) The SMARD profile that contains the aligned molecules are divided into 10 kb intervals (black vertical lines). The number of molecules with replication forks in each 10 kb interval in either the 3' to 5' or the 5' to 3' direction is calculated.
Top right) The numbers obtained from the Fig. on the left are represented as the percentage of molecules with replication forks (y-axis) within each 10 kb interval (x-axis). An interval with 10% or more of molecules with replication forks (interval 22) represents fork pausing in that interval.
Bottom right) This histogram is constructed from the schematic on the left. Percentage of IdU (first label) incorporation at each 10 kb interval is plotted along the DNA segment to generate the replication profile of the DNA segment shown at the left. This depicts the region that is replicated first within the DNA segment. The 10 kb intervals on the 3' end of the segment have the highest percentage of molecules with IdU incorporation indicating that the majority of molecules are replicated from the 3' to the 5' end and the 3' end is replicated before the 5' end in the majority of molecules.

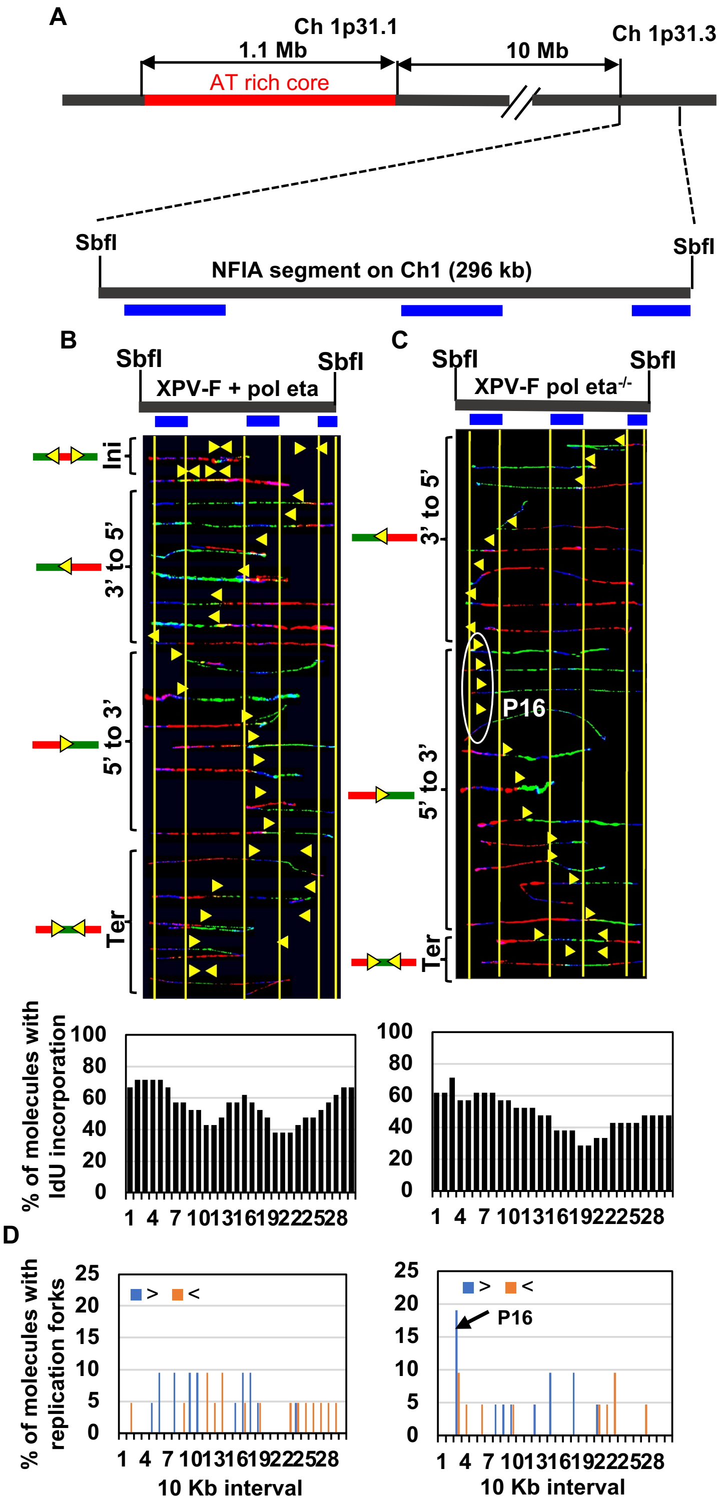

A
Ch 1p31.1
1.1 Mb
10 Mb
Ch 1p31.3
AT rich core
SbfI
SbfI
NFIA segment on Ch1 (296 kb)
B
C
SbfI
SbfI
XPV-F + pol eta
SbfI
SbfI
XPV-F pol eta-/-
Ini
3' to 5'
5' to 3'
Ter
P16
% of molecules with IdU incorporation
100
80
60
40
20
0
1 4 7 10 13 16 19 22 25 28
D
% of molecules with replication forks
25
20
15
10
5
0
> <
P16
10 Kb interval

Fig. S2. Replication is perturbed at the CFS on chromosome 1p31.3 in Pol eta deficient fibroblasts.

A) Locus map of NFIA segment with respect to AT-rich core of the CFS at chromosome 1p31.1.

B-C) Top: locus map of the NFIA segment with the location of the FISH probes. Middle: photomicrographs of labeled DNA molecules from XPV Pol eta$^{-/-}$ fibroblasts stably complemented with Pol eta (B) or not (C). Molecules are arranged as in Fig. 1. White oval (P16) indicates replication fork pausing. Bottom: the percentage of molecules with IdU incorporation at each 10 kb interval quantified from molecules above (middle).

D) The percentage of molecules with replication forks at each 10 kb interval in the NFIA segment, quantified from molecules in B-C. Replication forks progressing from 3’ to 5’ and 5’ to 3’ are denoted by orange < and blue > respectively. Black arrow (P16) indicates the most prominent pause peak along the NFIA segment and correspond to white oval in C.

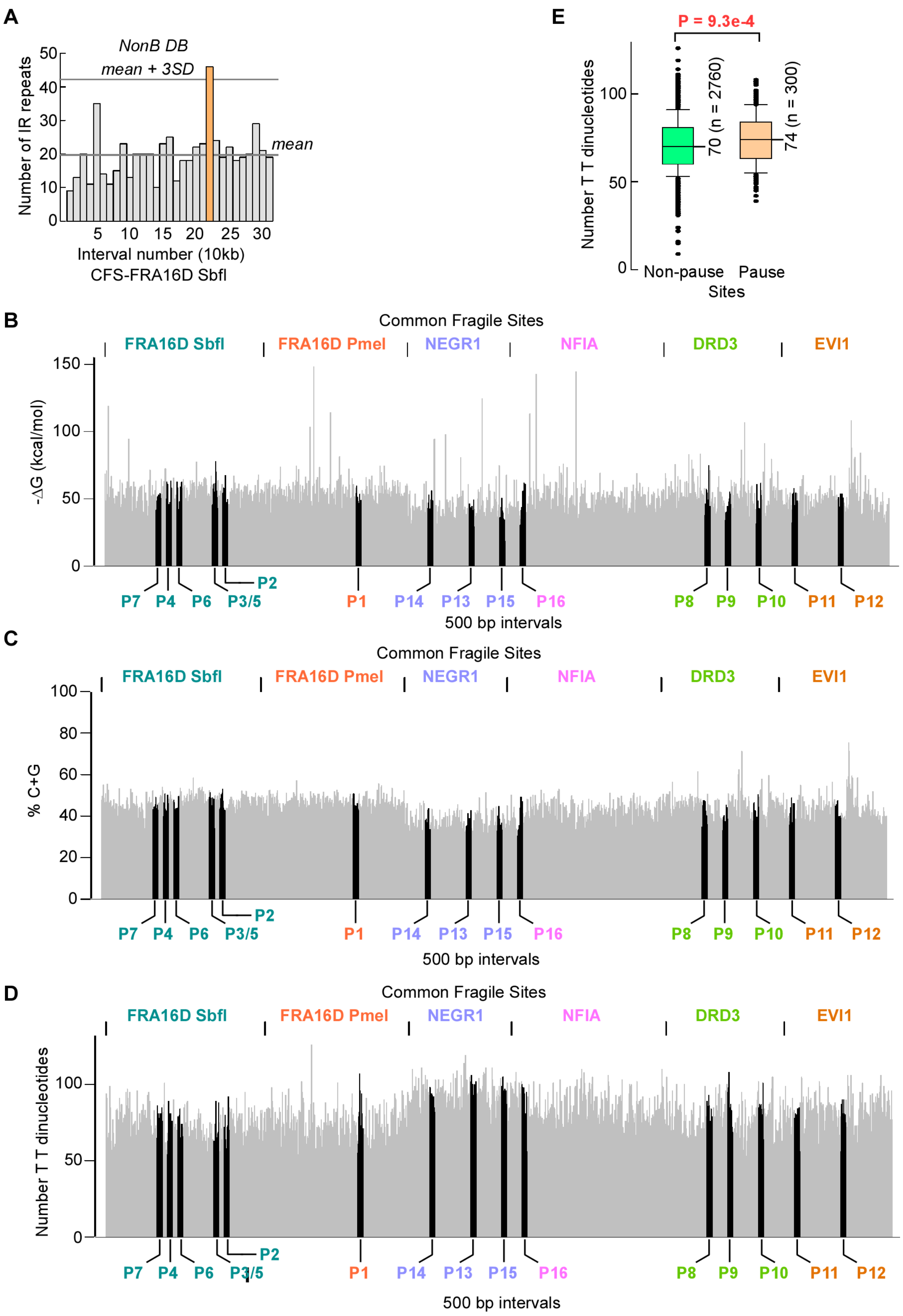
A
NonB DB
mean + 3SD
mean
Number of IR repeats
Interval number (10kb)
CFS-FRA16D SbfI
E
P = 9.3e-4
Number T T dinucleotides
70 (n = 2760)
74 (n = 300)
Non-pause
Pause
Sites
B
Common Fragile Sites
FRA16D SbfI
FRA16D PmeI
NEGR1
NFIA
DRD3
EVI1
-ΔG (kcal/mol)
P7 P4 P6 P3/5 P2 P1 P14 P13 P15 P16 P8 P9 P10 P11 P12
500 bp intervals
C
Common Fragile Sites
% C+G
500 bp intervals
D
Common Fragile Sites
Number T T dinucleotides
500 bp intervals

Fig. S3. Pausing is associated with non-B DNA structures and low C+G-content.

A) Bar plot of the number of inverted repeats found using non-B DB in each 10 kb interval of the CFS-FRA16D *SbfI* segment. *Orange*, interval 22 which contains pause site P3 in Pol eta deficient lymphoblasts and P5 in Pol eta deficient fibroblasts; *Reference lines*, mean and mean + 3 SDs for the number of non-B DNA-forming repeats in the total 31 intervals.

B) Bar plot of -ΔG (kcal/mol) values of the most stable mfold predicted structure for each 500-base interval of the combined six CFSs segments. Total number of intervals: pause sites, 300; non-pause sites, 2760.

C) Percentage of C+G, which is associated with the -ΔG values, in 500-base intervals for each of the six CFSs segments. Bar plot shows the percentage of C+G in each 500-base interval for the combined six CFSs segments. The total number of intervals examined was 3060 and includes 300 intervals for pause sites and 2760 intervals for non-pause sites.

D) Number of TT dinucleotides in 500 bp intervals for each of six CFSs segments. Bar plot shows the sum of TT dinucleotides in each 500-base interval for the combined six CFSs segments. The total number of intervals examined was 3060 and includes 300 intervals for pause sites and 2760 intervals for non-pause sites.

E) Box plot of data in (D), TT dinucleotides for the 500-base intervals comprising the combined six CFSs fragments. P-value from Mann-Whitney rank sum test.

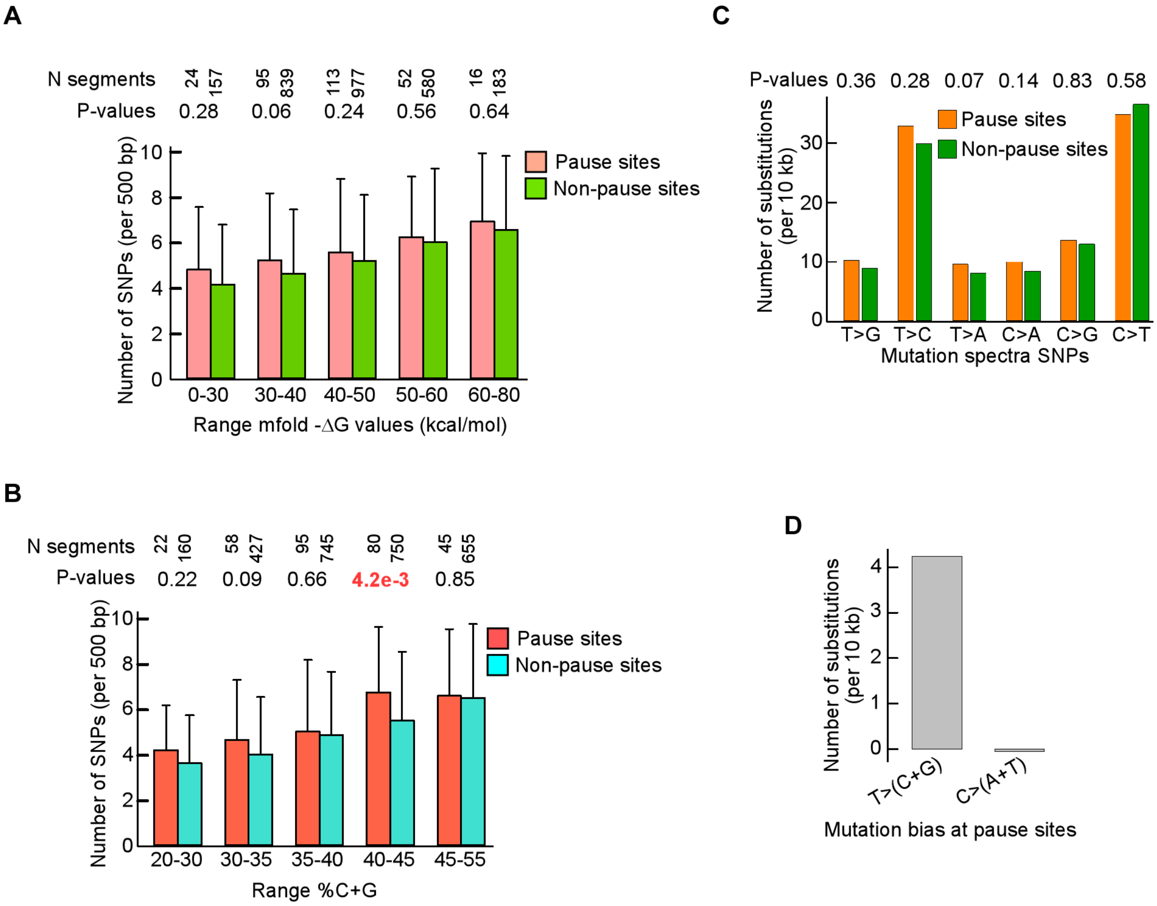

Fig. S4. Pausing is associated with increased genetic variation in the healthy human population.

A) Bar plot of number of SNPs per 500 bp as a function of -ΔG values from mfold. Ranges for -ΔG were divided so that every range contains at least 10 SNPs. Number of 500 bp intervals is shown on top. P-values from Welch's t-tests.

B) Bar plot of number of SNPs per 500 bp as a function of %C+G. Ranges for %C+G were divided so that every interval contains at least 10 SNPs. Number of 500 bp intervals is shown on top. P-values from Welch's t-tests.

C) Bar plot of mutational spectra for SNPs in the 10 kb intervals. P-values from Welch's t-tests.

D) Bar plot of net difference in the number of SNP substitutions at pause sites relative to non-pause sites. *Left*, T>(C+G) at pause sites minus T>(C+G) at non-pause sites); *right*, C>(A+T) at pause sites) minus C>(A+T) at non-pause sites.

A

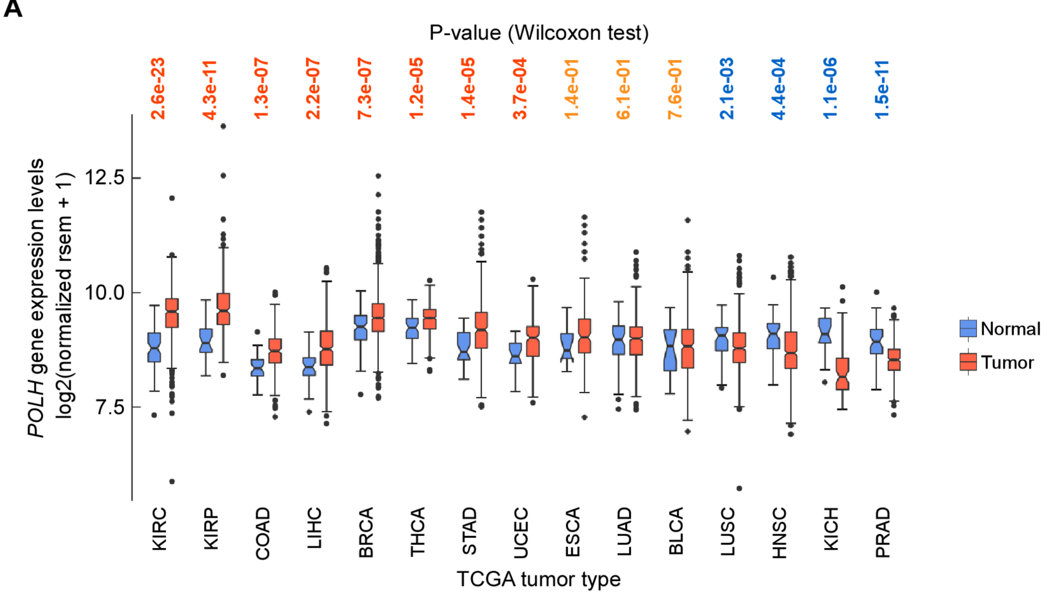

B

Tumor organ	Sig9+	Total	%Sig9+
Pancreas	404	915	44.1
Blood	233	626	37.2
Esophagus	226	615	36.7
Breast	595	1759	33.8
Prostate	267	791	33.8
Ovary	130	528	24.6
Skin	176	731	24.1
Kidney	189	788	24.0
Lymph_Nodes	8	37	21.6
Gall_Bladder	43	239	18.0
Bone	29	163	17.8
Brain	210	1273	16.5
Thymus	18	123	14.6
Colorectal	105	721	14.6
Bile_Duct	6	45	13.3
Liver	159	1303	12.2
Bone_Marrow	16	144	11.1
Pleura	7	82	8.53
Soft_tissue	13	237	5.5
Uterus	31	587	5.3
Stomach	22	448	4.9
Lung	53	1084	4.9
Testis	6	128	4.7
Adrenal_Gland	12	271	4.4
Head_and_neck	48	1258	3.8
Bladder	13	515	2.5
Eye	2	80	2.5
Cervix	5	289	1.7

C

Mutation spectra signature9+ patients

Subs.	Number	%
C>A	19431	20.2%
C>G	6920	7.2%
C>T	44109	45.8%
T>G	6070	6.3%
T>C	13743	14.3%
T>A	6039	6.3%
Total	96312	

Mutation spectra signature9- patients

Subs.	Number	%
C>A	37832	22.7%
C>G	13198	7.9%
C>T	85775	51.4%
T>G	7002	4.2%
T>C	16706	10.0%
T>A	6349	3.8%
Total	166862	

ATs to GCs = pos 20.6%; neg 14.2%
GCs to ATs = pos 66.0%; neg 74.1%

Fig. S5. High *POLH* gene expression and prevalence of signature 9 mutations in cancer.

A) *POLH* gene expression profile in tumors and matched controls. Tumor abbreviations are as follows: KIRC, kidney renal clear cell carcinoma; KIRP, kidney renal papillary cell carcinoma; COAD, colon adenocarcinoma; LIHC, liver hepatocellular carcinoma; BRCA, breast invasive carcinoma; THCA, thyroid carcinoma; STAD, stomach adenocarcinoma; UCEC, uterine corpus endometrial carcinoma; ESCA, esophageal carcinoma; LUAD, lung adenocarcinoma; BLCA, bladder urothelial carcinoma; LUSC, lung squamous cell carcinoma; HNSC, head and neck squamous cell carcinoma; KICH, kidney chromophobe; PRAD, prostate adenocarcinoma.

B) Prevalence of signature 9 mutations in cancer. Column 1, tissues affected by tumors; column 2, number of tumor samples with signature 9 mutations; column 3, total number of tumor samples; column 4, percent samples with signature 9 mutations.

C) Mutation spectra in signature 9+ and signature 9- samples from COSMIC.

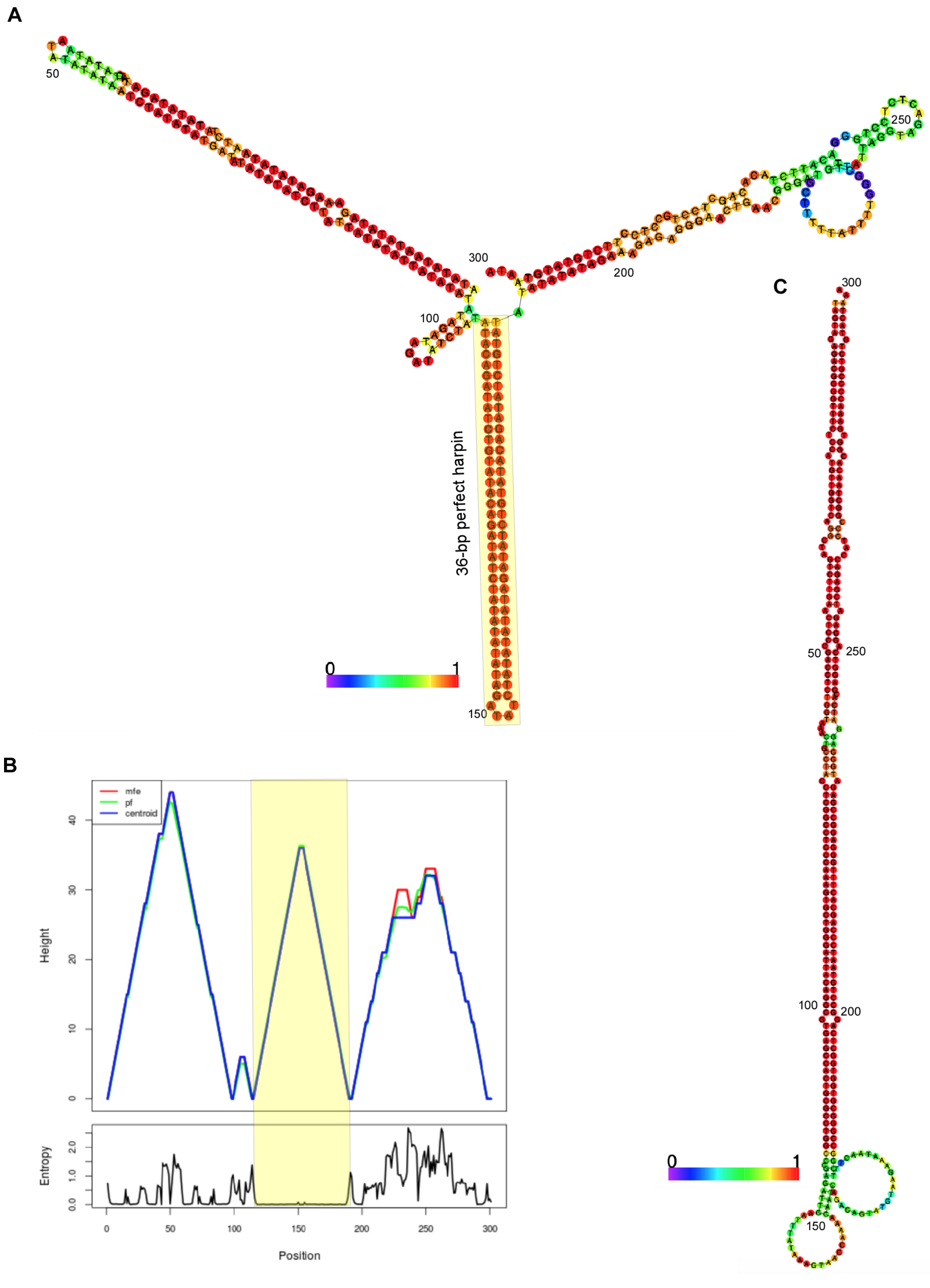
A
36-bp perfect harpin
50
100
150
200
250
300
0
1
B
mfe
pf
centroid
Height
Entropy
Position
C

Fig. S6. Predicted folding of local inverted repeats embedded within 300-base fragments.

A) Centroid drawing of base-pair probability of the secondary structure predicted from the long-inverted repeat in interval 22 of the CFS-FRA16D *SbfI* fragment created using RNAfold.

B) *Top*, mountain plot representation of the minimum free energy prediction (*red*), thermodynamic ensemble (*green*) and centroid structure (*blue*) of the sequence shown in panel A; peaks correspond to hairpin loops and slopes correspond to helices. *Bottom*, positional entropy along the DNA sequence. Both mountain plot and positional entropy plot were created using RNAfold.

C) Centroid drawing of base-pair probability of the secondary structure predicted from the longest inverted repeat scored by non-B DB in interval 14 of CFS-NEGR1 created using RNAfold.

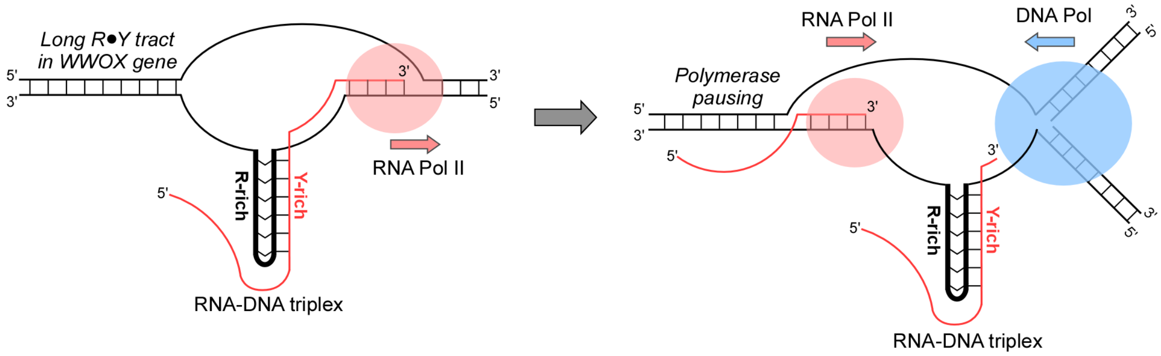

Fig. S7. Model for transcription-dependent replication pausing caused by hybrid RNA:DNA triplex structures formed by the TTCTT repeat in interval 19 of CFS-FRA16D *PmeI* fragment, which contains pause site P1 in Pol eta deficient lymphoblast. *Left*, accumulation of negative supercoiling behind the transcription bubble enables the DNA purine-rich strand (*thick line*) to fold onto itself and pair through reverse Hoogsteen hydrogen bonds, comprising A:A and G:G interactions (5), thereby forming a triplex structure with nascent RNA. *Right*, the persistent hybrid RNA:DNA triplex causes a topological barrier to converging transcription and replication. Other triplex isoforms are possible, including the Y-rich RNA sequence providing the third strand by binding to the template R-rich DNA sequence in the parallel orientation through Hoogsteen pairs. Reversing the pre-mRNA orientation is possible given the full palindromic nature of the 15 TTCTT repeats.

Table S1. Summary statistics of non-B DNA-forming repeats in CFS.
(Separate excel file)

Column A, interval number; column B, hg38 coordinates; column C, pause sites; columns D-H, number of repeats by non-B DB; columns I-M, total bases comprising each repeat type by non-B DB; columns N-R, number of repeats by custom scripts; columns S-W, total bases comprising each repeat type by custom scripts; columns X-Y; total number of bases for all repeats; columns Z-AD, longest repeats in CFS segment by custom scripts, unless indicated otherwise.

Table S2. Certain pause sites are associated with breakpoints of chromosomal rearrangements in cancer cells recorded in COSMIC database

(Separate excel file)

Table S3. Top inverted repeats from custom scripts

(Separate excel file)

List of inverted repeat motifs found with custom scripts. column A, CFS fragment and interval; column B, hg38 coordinate; column C, number of bp in each inverted repeat arm; column D, number of bases separating the repeats; columns E and F, sequence; column G, whether or not the repeats are in pause sites.

Table S4. Location of analyzed DNA segments in human genome assembly GRCh38, and corresponding PCR primers, and fosmids used to identify DNA segments.

Name of CFS DNA segment	Coordinates (GRCh38)		PCR Primer Sequence	Fosmids	Reference
	Start	End			
FRA16D *PmeI* 280 kb	Ch16: 78535654	Ch16: 78816150	5’GAGGCCTGGTGTATGCACTT<br>5’CTACAGACAGGCAGGCACAA	WI2-1680B6<br>WI2-933I24	(1)
FRA16D *SbfI* 305 kb	Ch16: 78815000	Ch16: 79120060	5’CCGATGCAACTGTCTGTCCT<br>5’TCCAACAACGGTCTCACCAG	WI2-644P9<br>WI2-2833E8<br>WI2-1310G20	(1)
DRD3 *PmeI* 231 kb	Ch3: 114081219	Ch3: 114312265	5’GCACTGGCCTTGCCATTCAC<br>5’TTTGGCAACTTAGGGGCCTTCA	WI2-2138I21<br>WI2-1358H20	This Study
EVI1 *PmeI* 213 kb	Ch3: 169526717	Ch3: 169739372	5’CCAGCTCCCAAGGAGGGAAT<br>5’CATCCGCAGTTCCAAAGGGC	WI2-516E5<br>WI2-3960G7	This Study
NEGR1 *SbfI* 199 kb	Ch1: 71989209	Ch1: 72188394	5’TGCTTCCCTGACTGTACCCC<br>5’ATGCCGCTTACCTATGGAGGG	WI2-2928A12<br>WI2-3681B5	This Study
NFIA *SbfI* 296 kb	Ch1: 61088223	Ch1: 61384788	5’AGCCTTGCCTCTGTATGCCC<br>5’GGCCTCGTATGTGCTACCTTGA	WI2-445F11<br>WI2-3221F8<br>WI2-1709C20	This Study

Supplemental References

1. A. Madireddy *et al.*, FANCD2 Facilitates Replication through Common Fragile Sites. *Molecular cell* **64**, 388-404 (2016).
2. W. C. Drosopoulos, S. T. Kosiyatrakul, C. L. Schildkraut, BLM helicase facilitates telomere replication during leading strand synthesis of telomeres. *The Journal of cell biology* **210**, 191-208 (2015).
3. W. C. Drosopoulos *et al.*, TRF2 Mediates Replication Initiation within Human Telomeres to Prevent Telomere Dysfunction. *Cell reports* **33** (2020).
4. A. Bacolla, J. A. Tainer, K. M. Vasquez, D. N. Cooper, Translocation and deletion breakpoints in cancer genomes are associated with potential non-B DNA-forming sequences. *Nucleic acids research* **44**, 5673-5688 (2016).
5. M. D. Frank-Kamenetskii, S. M. Mirkin, TRIPLEX DNA STRUCTURES. *Annual review of biochemistry* **64**, 65-95 (1995).
